# Functional Changes in GABA and Glutamate during Motor Learning

**DOI:** 10.1523/ENEURO.0356-20.2023

**Published:** 2023-02-23

**Authors:** Tiffany K. Bell, Alexander R. Craven, Kenneth Hugdahl, Ralph Noeske, Ashley D. Harris

**Affiliations:** 1 Department of Radiology, University of Calgary, Calgary, Alberta T2N 1N4, Canada; 2 Hotchkiss Brain Institute, University of Calgary, Calgary, Alberta T2N 1N4, Canada; 3 Alberta Children's Hospital Research Institute, University of Calgary, Calgary, Alberta T2N 1N4, Canada; 4 Department of Biological and Medical Psychology, University of Bergen, NO-5020 Bergen, Norway; 5 Department of Clinical Engineering, Haukeland University Hospital, N-5021 Bergen, Norway; 6 Division of Psychiatry, Haukeland University Hospital, N-5021 Bergen, Norway; 7 Department of Radiology, Haukeland University Hospital, N-5021 Bergen, Norway; 8 NORMENT Center for the Study of Mental Disorders, Oslo University Hospital HF, N-0450 Bergen, Norway; 9 GE Healthcare, 12277 Berlin, Germany

**Keywords:** functional magnetic resonance spectroscopy (fMRS), GABA, GABA editing, glutamate, MEGA-PRESS, motor learning

## Abstract

Functional magnetic resonance spectroscopy (fMRS) of GABA at 3 T poses additional challenges compared with fMRS of other metabolites because of the difficulties of measuring GABA levels; GABA is present in the brain at relatively low concentrations, and its signal is overlapped by higher concentration metabolites. Using 7 T fMRS, GABA levels have been shown to decrease specifically during motor learning (and not during a control task). Though the use of 7 T is appealing, access is limited. For GABA fMRS to be widely accessible, it is essential to develop this method at 3 T. Nine healthy right-handed participants completed a motor learning and a control button-pressing task. fMRS data were acquired from the left sensorimotor cortex during the task using a continuous GABA-edited MEGA-PRESS acquisition at 3 T. We found no significant changes in GABA+/tCr, Glx/tCr, or Glu/tCr levels in either task; however, we show a positive relationship between motor learning and glutamate levels both at rest and at the start of the task. Though further refinement and validation of this method is needed, this study represents a further step in using fMRS at 3 T to probe GABA levels in both healthy cognition and clinical disorders.

## Significance Statement

GABA is the major inhibitory neurotransmitter in the brain and plays a key role in motor learning. Functional, noninvasive measures of GABA in humans *in vivo* is desirable; however, there has been little development of GABA functional magnetic resonance spectroscopy (fMRS) because of the challenges of measuring GABA. This study investigates the use of GABA-editing (Mescher-Garwood point resolved spectroscopy, MEGA-PRESS) at 3 T to acquire functional measures of GABA and glutamate levels during a behavior task. Our study highlights some of the issues facing the fMRS literature and can be used to guide future studies investigating GABA and glutamate levels simultaneously during motor learning.

## Introduction

Proton magnetic resonance spectroscopy (MRS) is a noninvasive technique using a magnetic resonance imaging (MRI) scanner which measures *in vivo* metabolite levels, including the inhibitory neurotransmitter GABA and the excitatory neurotransmitter glutamate. Typically, data are acquired over a static period of several minutes during an “at-rest” state; however, this provides limited information regarding cerebral function.

Functional MRS (fMRS) involves taking multiple spectra during a task to provide a dynamic measure of neurochemistry changes in response to stimuli (Jelen et al., 2018). Of the relatively few fMRS studies conducted, most focus on glutamate because of its prominent role in neural signaling and ease of measurement; glutamate can be measured relatively easy at 3 T using a standard point resolved spectroscopy (PRESS) sequence with sufficient signal-to-noise ratio (SNR) obtained from 30 s of measurements (Woodcock et al., 2018; for review of fMRS of glutamate, see Mullins, 2018, Stanley and Raz, 2018).

GABA also has a prominent role in neural signaling, but fMRS of GABA is less common because of the challenges associated with even resting measurements. GABA is present in the brain at relatively low concentrations, and its signal is overlapped by higher-concentration metabolites. One solution is to use a high magnetic field strength, which increases the SNR and spectral resolution, facilitating metabolite separation and quantification of lower-concentration metabolites (Pradhan et al., 2015). At 7 T, Kolasinski et al. (2019) used an analysis consisting of six, 6 min blocks to show an ∼20% decrease in GABA during a motor learning task. Though the use of 7 T is appealing, access is limited. For fMRS of GABA to be accessible in both research and clinical settings, it is essential to develop this method at 3 T, the more commonly used field strength.

One approach to resolve GABA at 3 T is to use Mescher-Garwood PRESS (MEGA-PRESS; Mescher et al., 1998). Briefly, J-coupling within the GABA molecule is exploited to modulate the GABA signal without affecting the other metabolites in half of the acquisition. A difference spectrum is generated in which the overlapping resonances have been removed to facilitate quantification of GABA. For a complete review of this approach see Mullins et al. (2014) and Harris et al. (2017).

Floyer-Lea et al. (2006) used MEGA-PRESS at 3 T to also show an ∼20% GABA decrease in the sensorimotor cortex during motor learning; however, this finding has yet to be replicated at 3 T. Additionally, both the study by Floyer-Lea et al. (2006; three 8 min blocks) and the study by Kolasinski et al. (2019; six 6 min blocks) had very low temporal resolution, severely limiting the study of metabolite dynamics. Furthermore, glutamate changes often occur on a much faster timescale, and thus may not be detected using long blocks. Chen et al. (2017) used a sliding-window analysis to show an almost immediate increase in glutamate at 7 T in response to hand clenching, whereas GABA changes occurred on a much slower timescale (3–5 min). Therefore, a block-averaged analysis may obscure metabolite changes on different timescales and will be biased toward changes on a similar timescale as the block duration. Indeed, Kolasinski et al. (2019) found no changes in glutamate levels using a block-average analysis; however, the findings by Chen et al. (2017) suggest glutamate may have increased in response to hand movement during the task.

The aim of this study was to develop and validate fMRS of GABA and glutamate using a MEGA-PRESS acquisition at 3 T and a sliding-window analysis. Participants performed a serial reaction time task twice in a repeated-measures design, once with a learning condition and once with no learning (movement condition). MEGA-PRESS data were continuously acquired throughout the task to allow for analysis in both a block-averaged and an event-related design. Glutamate was quantified from the OFF sub-spectra of the MEGA-PRESS data, which were recently shown to be in reasonable agreement with those for glutamate quantified from PRESS data when acquiring data from the sensorimotor cortex (Bell et al., 2020).

### Aim 1

Aim 1 was to quantify GABA and glutamate changes in response to a motor learning task at 3 T using a MEGA-PRESS acquisition and a block-averaged analysis. GABA was expected to decrease in response to a motor learning task, but not in response to movement on its own. Glutamate changes were not expected.

### Aim 2

Aim 2 was to implement a sliding-window analysis of GABA and glutamate changes during a motor learning task. GABA was expected to decrease over the course of minutes and remain decreased during the motor learning task. By contrast, more rapid, short-term glutamate increases were expected in both the motor learning and the control-movement task.

## Materials and Methods

### Sample characteristics

Nine right-handed participants of either sex who were 18–40 years of age were recruited. Participants were eligible for inclusion if they met the standard MRI safety criteria, were right handed (self-reported), had no current medical conditions, and no neurologic or psychiatric conditions either currently or previously. As participants needed to see the screen, they were required to have normal or normal-corrected vision.

### Sample size calculation

Kolasinski et al. (2019) report partial η^2^ = 0.334. While only four participants would be required to detect an effect of this magnitude (90% power, α = 0.05), as a more conservative effect size we assume a partial η^2^ = 0.2 to determine seven participants are required. The recruitment of nine participants allows 20% data loss because of poor data quality over the two sessions. As a reference, 14 participants would be required to detect an effect size of partial η^2^ = 0.1. We report this calculation in the case that this study produces negative results, so that these will be informative.

### Experimental procedures

#### Design

Participants were scanned twice, at the same time of day, 1 week apart in a crossover design, performing both the learning and movement conditions. The order of the conditions was counterbalanced across participants.

#### Serial reaction time task

A serial reaction time task was used for both the learning and the movement conditions, as described in the study by Kolasinski et al. (2019). Participants responded with their right hand using a four-button response box. Briefly, on the screen each finger was represented by one of four horizontal lines. In each trial, one of the horizonal lines was replaced with an asterisk for 150 ms. Participants were to press the corresponding button as quickly as possible in response to this cue. Forty-eight trials were presented with an interstimulus interval of 850 ms between cues. This was repeated six times within each epoch, with a rest period of 15 s at the end of each epoch. There were six epochs in total, each lasting ∼5 min.

In the learning condition, a 16-item sequence was repeated three times per epoch, and participants were explicitly informed to expect a repeating sequence. Learning was assessed by response time, and a decrease in response time indicated that a participant had learned the sequence. Task accuracy was assessed based on the number of correct presses per epoch. In the movement condition, cues were pseudorandomized to produce a different sequence of 48 cues in each epoch, and the number of button presses for each finger was matched to the learning task. Participants were explicitly told not to expect a sequence.

#### MRS data acquisition

**Figure 1.**
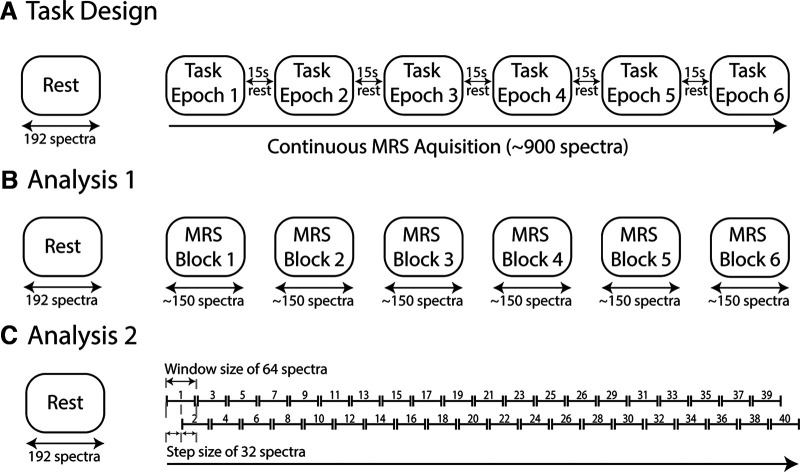
***A***, Task design. The task consists of 6 epochs, each lasting ∼5 min, with a 15 s rest period in between. Each epoch consists of 48 trials in which participants are asked to press a button as quickly as possible in response to a cue. MRS data were continuously acquired during the task using a MEGA-PRESS sequence. ***B***, Analysis 1. Spectra was averaged into blocks corresponding to the length of each task epoch, creating 6 averages of ∼150 spectra. ANOVAs were used to compare metabolite levels across blocks. ***C***, Analysis 2. Spectra were analyzed using a sliding-window approach with a window size of 64 and a step size of 32, providing ∼40 timepoints.

Data were collected on a 3 T scanner (model MR750w, GE Healthcare) with a 32-channel head coil. A T_1_-weighted image (BRAVO) was acquired for voxel placement and tissue segmentation [repetition time (TR) = 7.3 ms; echo time (TE) = 2.7 ms; 1 mm^3^ isotropic voxels; flip angle = 10°; inversion time = 600 ms] and tissue segmentation. The MRS voxel (2.5 × 2.5 × 2.5 cm^3^) was placed in the left sensorimotor cortex, centered at the hand-knob of the motor cortex, and rotated such that the coronal and sagittal planes aligned with the cortical surface (Yousry et al., 1997). For each participant, the voxel mask generated from session 1 was used to guide placement of the voxel in session 2. All MRS data were acquired using MEGA-PRESS (14 ms editing pulses; ON = 1.9 ppm; OFF = 7.46 ppm; TR = 1800 ms; TE = 68 ms; 4096 data points sampled at 5 kHz; eight-step phase cycle). MRS data were acquired before the task to provide an “at-rest” measure (192 averages; Mikkelsen et al., 2018a), and then continuously acquired throughout the task (∼30 min; Fig. 1). When using this sequence, the GABA signal is contaminated by ∼50% macromolecules; henceforth, GABA will be referred to as GABA+, to represent GABA + macromolecules. Though sequence modifications are available to suppress the macromolecule signal, this results in a reduction in the signal-to-noise ratio and typically requires an increased number of spectral averages for quantification of GABA (Harris et al., 2015b). Additionally, macromolecule suppressed acquisitions are also more sensitive to frequency drift and motion artifacts, which may introduce errors during the relatively long acquisition (Edden et al., 2016; Mikkelsen et al., 2017). Therefore, a GABA+ sequence was chosen for this study.

### Analysis pipeline

#### Task data analysis

Task accuracy was calculated as the percentage of correct button presses per block. The median reaction time for each of the six epochs was calculated (using correct responses only) per subject, per condition. The median reaction time was chosen for analysis over the mean to reduce the influence of outliers, such as slow responses because of attention effects.

#### MRS data analysis

Task MRS data were averaged using Gannet 3.2 (Edden et al., 2014) in the following two ways. First, spectra were averaged per subject into blocks corresponding to the length of each task epoch, creating six averages of ∼150 spectra (Fig. 1*B*). Second, task data were averaged into windows of 64 spectra using a sliding-window approach with a step size of 32, providing ∼40 timepoints (Fig. 1*C*). Though GABA-edited MRS data typically consist of an average of 200–300 spectra, data acquired from an average of 64 spectra in the sensorimotor cortex has been shown to be of sufficient quality for metabolite quantification (Mikkelsen et al., 2018a).

All data were preprocessed in Gannet 3.2 including coil combination, frequency and phase correction, and removal of motion-corrupted spectra. GABA+ was quantified using Gannet 3.2 (Edden et al., 2014), and the α-correction was applied, which assumes twice as much GABA in gray matter as in white matter (Harris et al., 2015a). Glutamate was quantified from the OFF subspectra using LCModel (Provencher, 2001). Because of the challenges of separating the glutamate signal from the signal of glutamine, its precursor, glutamate is reported as both glutamate and Glx (the sum of glutamate and glutamine). GABA+ and glutamate are referenced to creatine (tCr) from the edit-off subspectra, consistent with quantification described in the study by Kolasinski et al. (2019). Additionally, to confirm that any changes seen are specific to GABA+ and glutamate, we also referenced to N-acetyl aspartate (NAA) from the edit-off subspectra. The data quality of each averaged block and window was visually assessed. Data from each block or window was excluded if the Cramér–Rao lower bounds (CRLBs) of glutamate exceeded 50%, the SNR (NAA peak amplitude divided by the SD of the noise) was <20, or the NAA linewidth exceeded 13 Hz (Dhamala et al., 2019; Maudsley et al., 2021).

#### Consideration of T2 effects

A potential confound of fMRS is the effect of the blood oxygenation level-dependent (BOLD) signal, which has been shown to narrow task-related spectral linewidths in the motor cortex by 0.25 Hz at 7 T, though has yet to be reported at 3 T (Stanley and Raz, 2018). To determine whether the BOLD signal has an effect on fMRS in the motor cortex at 3 T across time, the resting data averaged across all participants were used as a reference. Each functional block, also averaged across all participants, was compared with the baseline by subtracting the baseline from the functional block. Residuals in the NAA and Cr regions of the group difference spectra would indicate BOLD effects. If BOLD effects are present, the optimal line-broadening value was determined by minimizing the residual NAA and Cr signals in the group difference spectra and was applied before quantification (Zhu and Chen, 2001; Mangia et al., 2007; Schaller et al., 2013; Boillat et al., 2020).

### Statistical analysis

Statistical analysis was conducted in R (R Core Team, 2019). As described in the study by Kolasinski et al. (2019), behavioral data were analyzed using a two-way repeated-measures ANOVA to compare changes in reaction time across the tasks, with condition (learning or movement) and time (epochs 1–6) as within-participant factors. Significant interactions were followed-up by simple main-effects analyses within each experimental group. *Post hoc* paired-samples *t* tests were used to compare the median reaction time of each epoch with that of epoch 1. An interaction effect was expected between condition and time, with a significant change in reaction time seen in the learning condition, but not the movement condition.

#### Aim 1: block-averaged analysis

Following the analysis described in the study by Kolasinski et al. (2019), a two-way mixed ANOVA was used to compare changes in GABA+ levels (quantified from the block-averaged spectra) across tasks (as with the behavioral data). Significant interactions were followed up by simple main-effects analyses within each experimental group. *Post hoc* paired-samples *t* tests were used to compare GABA+ levels within each epoch with epoch 1. Correlation analyses were used to assess the relationship between GABA+ levels and learning (quantified as the percentage change in reaction time). The same analysis was applied to glutamate levels.

#### Aim 2: sliding-window analysis

A mixed-effects model was used to compare changes in GABA+ levels across tasks, with condition treated as a fixed effect. Significant interactions were followed up by simple main-effects analyses within each experimental group. Dunnett’s test was used to determine the critical distance between GABA+ levels at baseline with each subsequent window. Differences above this level were considered significant. The same analysis was applied to glutamate levels.

Additionally, linear and nonlinear fitting was used to examine the time course of GABA+ and glutamate changes. *R*^2^ and mean squared error were used to quantify the fit of the different mathematical models. The relationship between the percentage change in and the rate of change of neurochemical levels and learning (quantified as the percentage change in reaction time) was assessed using correlation analyses.

### Change to registered report

We would like to report a minor change to the analyses. To determine the effects of the BOLD signal on linewidth, we originally stated that we would subtract the group-averaged resting data from the group-averaged functional data for each block. Instead, we used the tool op_matchLW from the FID-A toolbox (Simpson et al., 2017) to measure the difference in linewidth between the group-averaged resting data from the group-averaged functional data for each block, removing any subjectivity from the procedure. Data were aligned before averaging using the op_alignAverages function. Scripts for this procedure can be found at https://osf.io/qja95/.

## Results

This study was conducted as a registered report. The approved Stage 1 protocol can be found at the Open Science Framework at https://osf.io/zepbu/. Following in-principle acceptance, data were collected from nine participants (four males, five females; mean age, 27 years). Anonymized subject data, analysis scripts, and line-broadening values applied in each analysis can be found at https://osf.io/qja95/.

### Data quality

**Figure 2.**
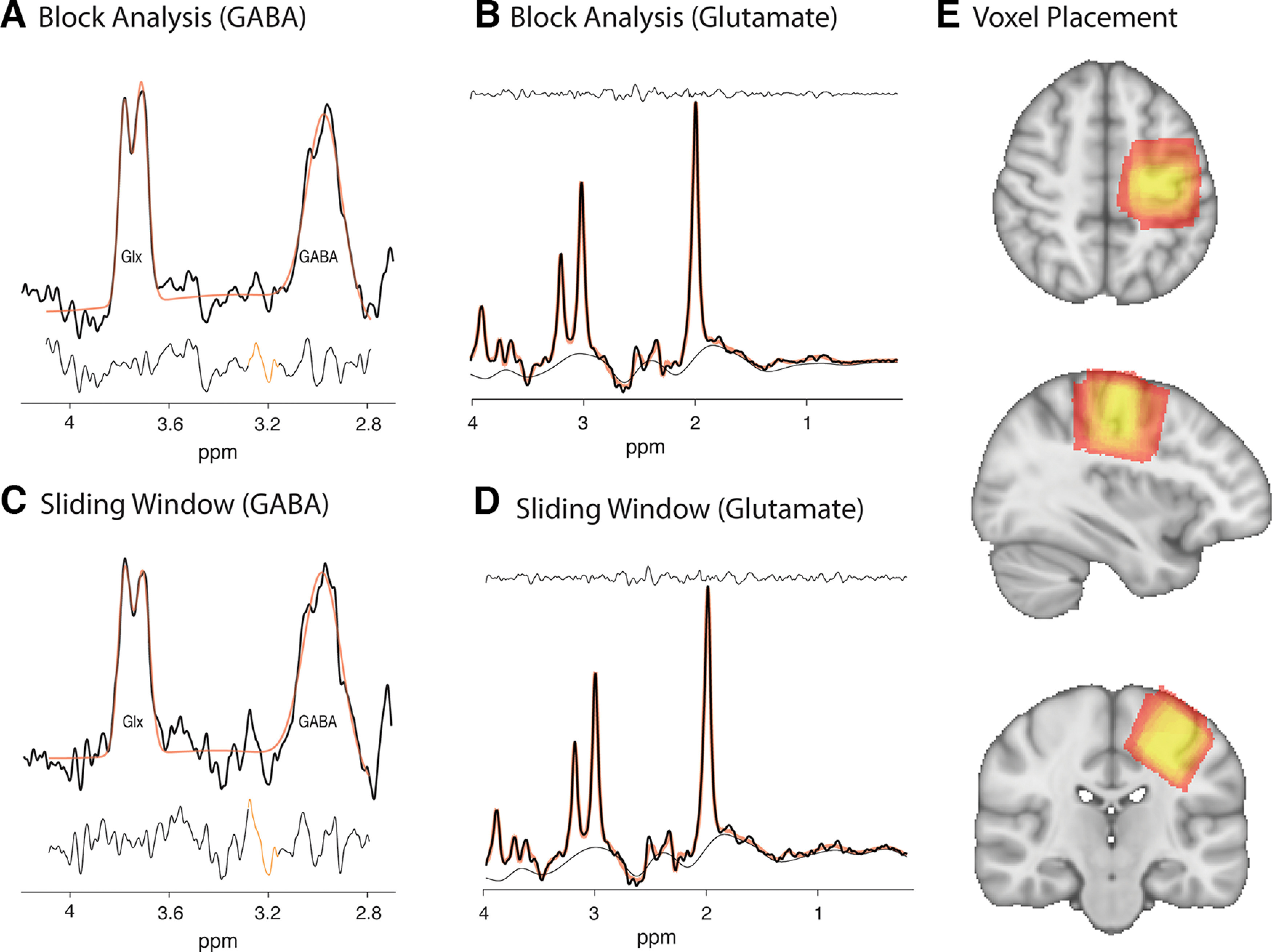
***A***, Example of GABA+ data from the block analysis [number of signal averages (NSA) = 184]. ***B***, Example of glutamate data obtained from the OFF sub-spectra from the block analysis (NSA = 184). ***C***, Example of GABA+ data from the sliding-window analysis (NSA = 64). ***D***, Example of glutamate data obtained from the OFF sub-spectra from the sliding-window analysis (NSA = 64). ***E***, Heatmap of voxel placement, yellow shows areas of high overlap.

**Figure 3.**
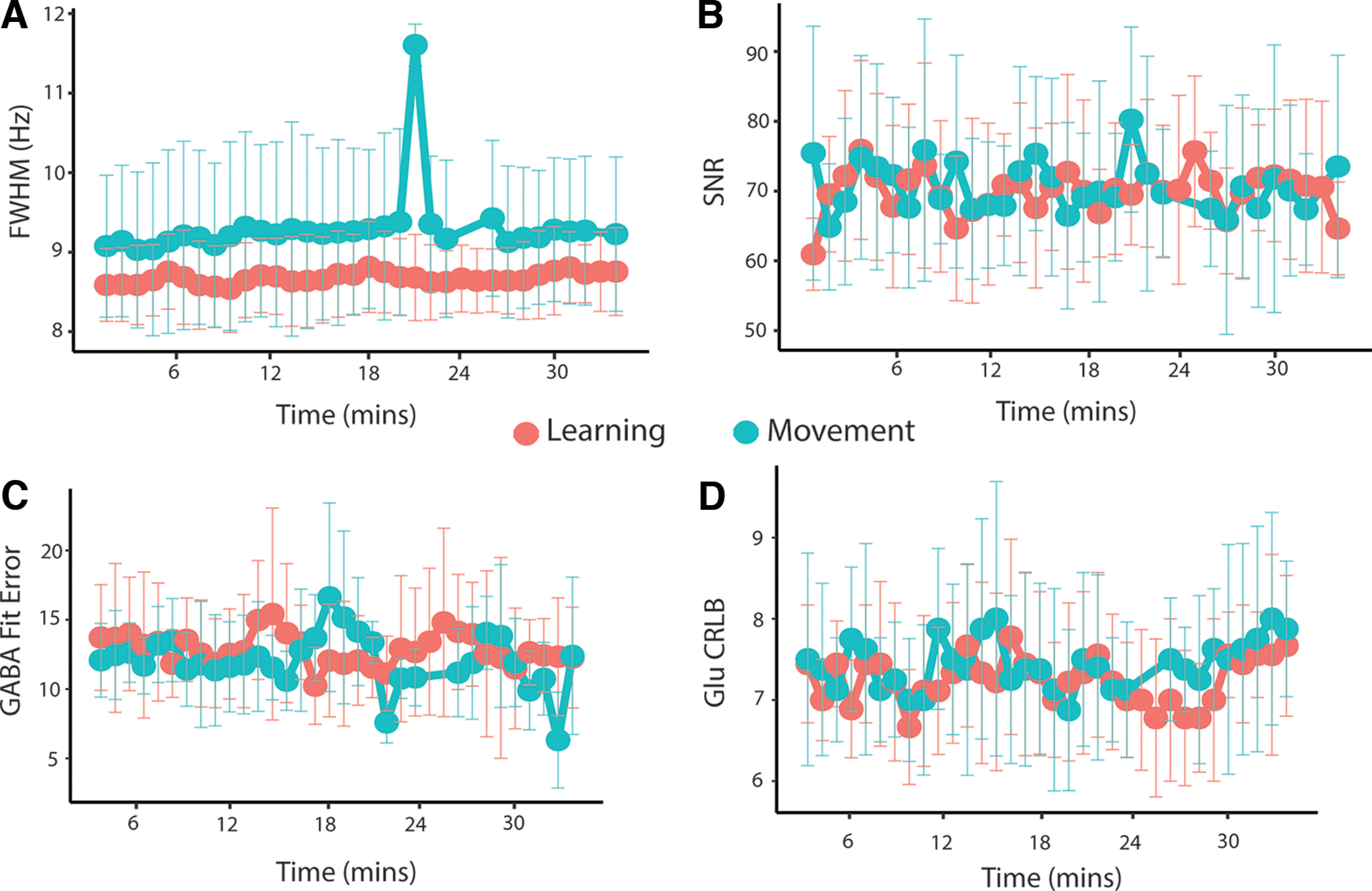
Quality metrics for sliding-window analysis. ***A***, Mean NAA FWHM in hertz. ***B***, Mean NAA SNR. Error bars represent the SD. ***C***, Mean GABA+ fit error (%) calculated in Gannet. ***D***, Mean glutamate CRLB calculated in LCModel.

**Figure 4.**
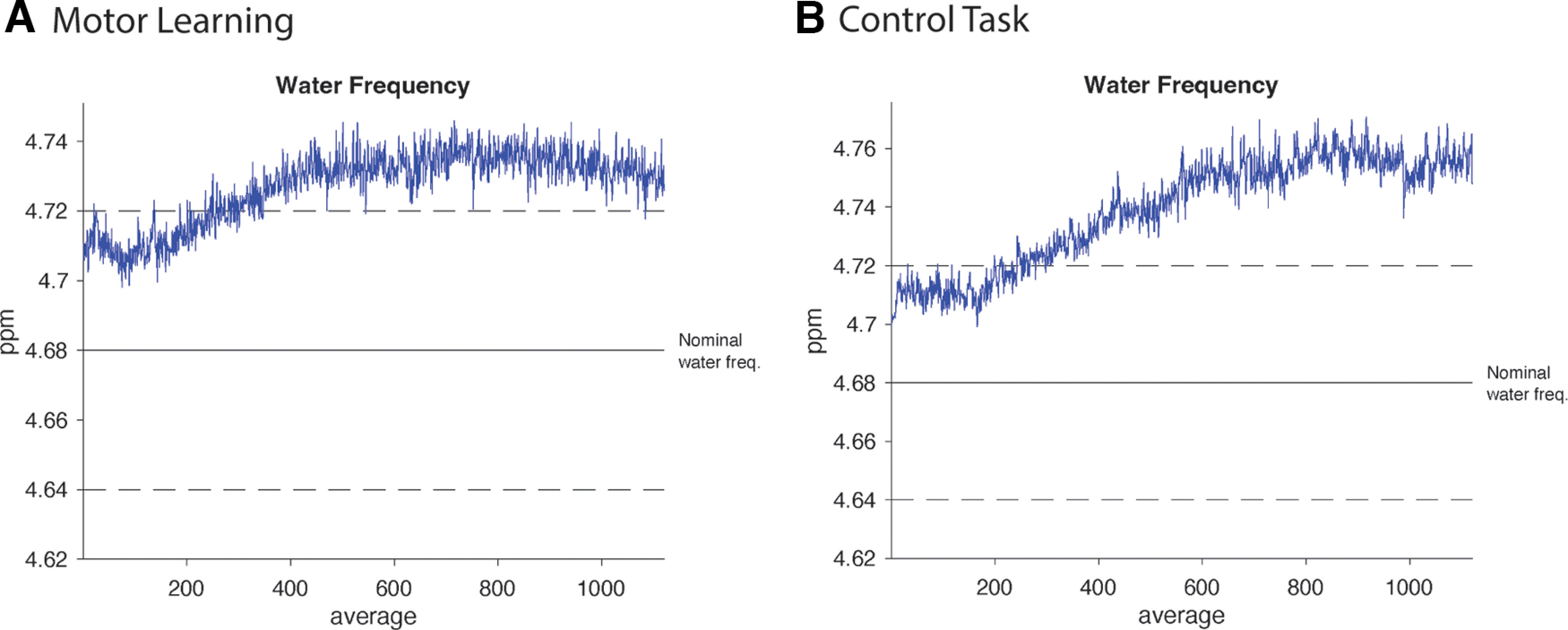
Example of frequency drift across the full data acquisition of each task from a single subject. ***A***, Frequency drift during the motor learning task. ***B***, Frequency drift during the control task.

Figure 2 shows example data from a single subject for both the block and sliding-widow analyses. Table 1 and Figure 3 summarize quality metrics averaged over the entire group for the block and sliding-window analyses, respectively. Linewidth and SNR were calculated using tools from the FID-A toolbox. Linewidth was calculated as the full-width at half-maximum (FWHM) of the NAA peak using the tool op_getLW. SNR was calculated as the amplitude of the NAA peak (1.8–2.2 ppm) divided by the SD of the noise (−2 to 0 ppm) using the tool op_getSNR. Table 2 and Figure 3 show fit metrics averaged over the entire group for the block and sliding-window analyses, respectively. Figure 4 shows examples of frequency drift across the full data acquisition of each task in a single participant.

**Table 1:** Quality metrics for block analysis

	NAA FWHM (Hz)		NAA SNR	
	Motor learning	Control task	Motor learning	Control task
Block 1	8.49 (0.46)	8.92 (1.00)	115.11 (17.64)	117.90 (21.92)
Block 2	8.45 (0.51)	9.03 (1.11)	108.42 (14.05)	117.29 (18.80)
Block 3	8.52 (0.54)	9.08 (1.17)	110.47 (17.37)	112.44 (15.44)
Block 4	8.53 (0.48)	9.09 (1.02)	112.48 (17.03)	113.39 (22.91)
Block 5	8.52 (0.45)	9.05 (0.98)	113.39 (12.41)	107.16 (21.97)
Block 6	8.55 (0.47)	9.05 (0.95)	106.73 (15.37)	109.03 (23.36)

Values are mean (SD).

**Table 2:** Fit metrics for block analysis

	GABA fit error		Glutamate CRLB	
	Motor learning	Control task	Motor learning	Control task
Block 1	8.94 (2.61)	8.71 (1.29)	6.44 (1.01)	6.50 (0.53)
Block 2	8.79 (1.76)	7.66 (2.23)	6.22 (0.83)	6.00 (0.76)
Block 3	9.06 (2.21)	9.03 (1.41)	6.78 (0.44)	6.63 (0.74)
Block 4	7.66 (1.39)	7.60 (0.90)	6.67 (0.71)	6.63 (0.74)
Block 5	9.38 (2.64)	8.62 (1.48)	6.44 (0.53)	6.75 (0.46)
Block 6	8.26 (1.80)	8.29 (3.46)	6.44 (0.88)	7.00 (0.53)

Values are mean (SD).

The MRS data from the control task for one subject was removed because of technical issues during acquisition. In the sliding-window analysis, MRS data from all subjects for windows 24 (spectra 737–800; approximately minute 22) and 25 (spectra 769–832; approximately minute 23) and MRS data from three subjects from window 21 (spectra 641–704, approximately 19 min) were removed from the control task because of a NAA linewidth of >13 Hz.

To confirm that any changes seen are specific to GABA+ and glutamate, we performed a secondary analysis with metabolites referenced to NAA from the edit-off sub-spectra. These results were in line with tCr-referenced-referenced values and therefore are not reported further.

### Reaction time

**Figure 5.**
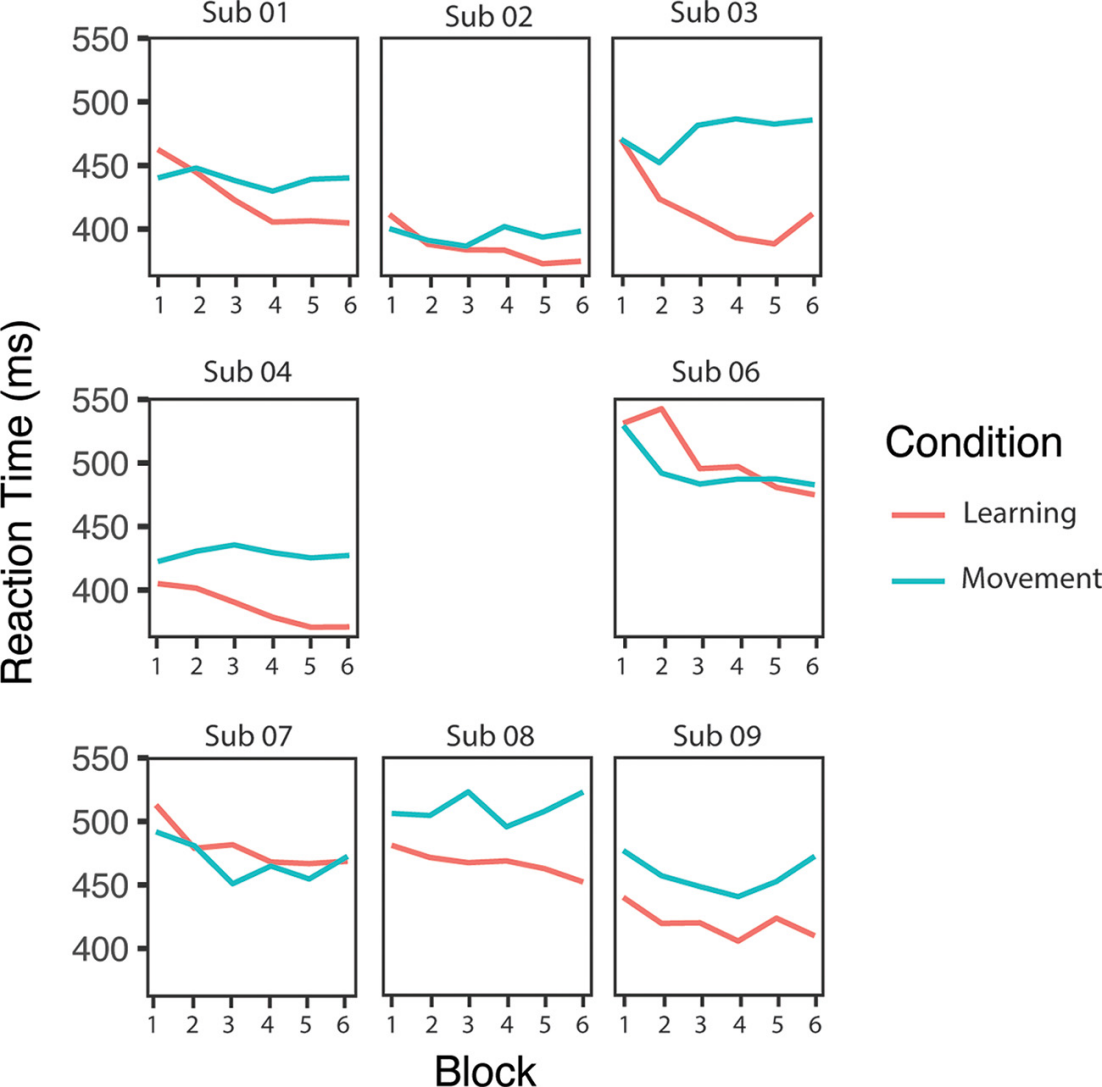
Reaction time for each task block for each individual participant. Note: subject (Sub) 05 was removed because their button presses often preceded the queue.

Figure 5 shows individual timecourses for each participant’s reaction time. One subject was removed from reaction time analyses as their button presses often preceded the cue. Removal of this subject from GABA and glutamate analyses had no effect on the overall results; therefore, this subject was left in for the metabolite analyses.

**Figure 6.**
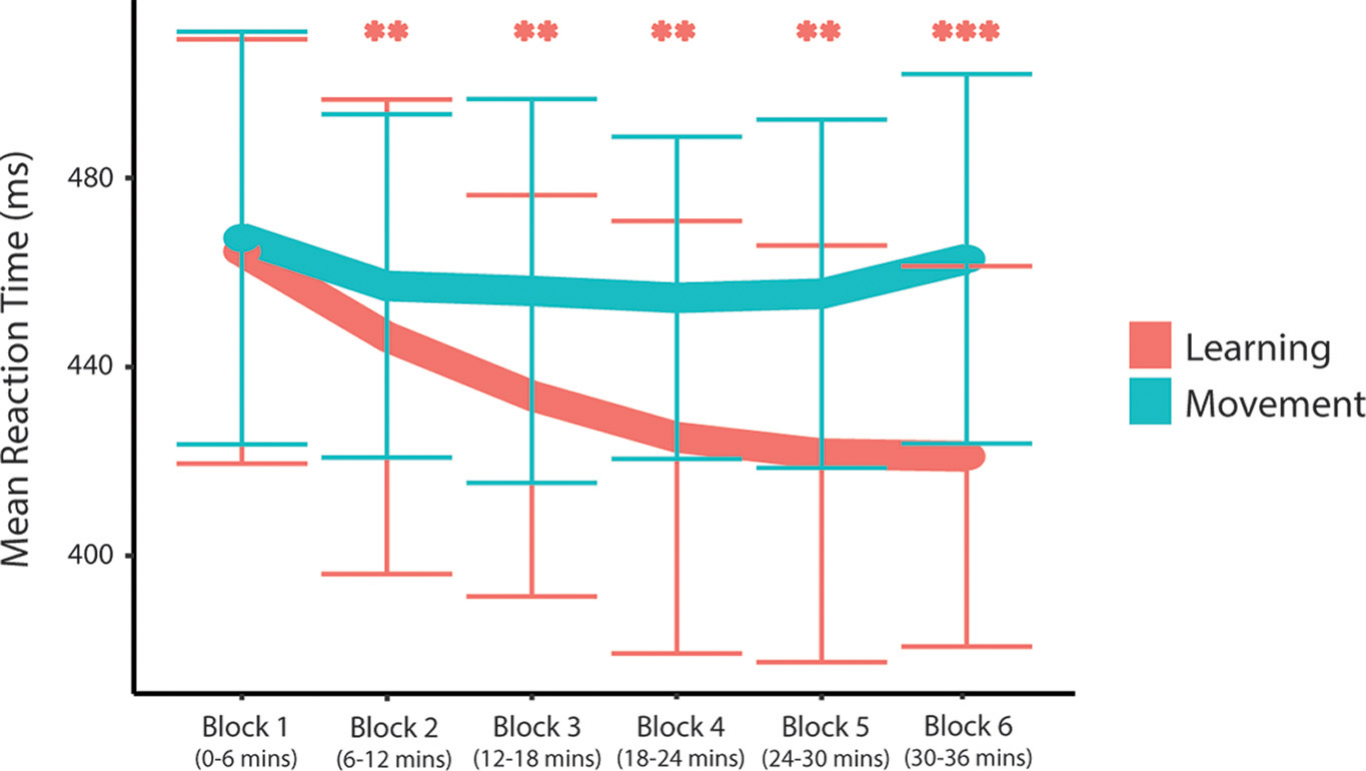
Mean reaction time for each block throughout the task. Error bars represent the SD. Comparisons made to block 1: *p < 0.05, **p < 0.01, ***p < 0.001. All *p*-values are Bonferroni adjusted.

The two-way repeated-measures ANOVA examining reaction time showed a significant effect of condition (*F*(1.0,7.0) = 6.4, *p* = 0.04, η^2^ = 0.48), a significant effect of time (*F*(5.0,35.0) = 13.5, *p* < 0.001, η^2^ = 0.66) and a significant time by condition interaction effect on reaction time (*F*(2.1,14.8) = 6.8, *p* = 0.007, η^2^ = 0.49). Simple main-effects analyses showed a significant effect of time in the learning condition only (learning: *F*(2.3,16.4) = 19.8, *p* < 0.001, η^2^ = 0.74, Bonferroni-adjusted *p*-value; movement: *F*(5,35) = 1.5, *p* = 0.42, η^2^ = 0.18, Bonferroni adjusted *p*-value). Compared with block 1 (ending at 6 min), *post hoc* paired-samples *t* tests showed a significant reduction in reaction time for blocks 2, 3, 4, 5, and 6 (i.e., ending at 12, 18, 24, 30, and 36 min into the protocol; *p* < 0.01, Bonferroni adjusted *p*-value) in the learning condition only, with no significant change in reaction time in the movement group (Fig. 6).

### GABA

**Figure 7.**
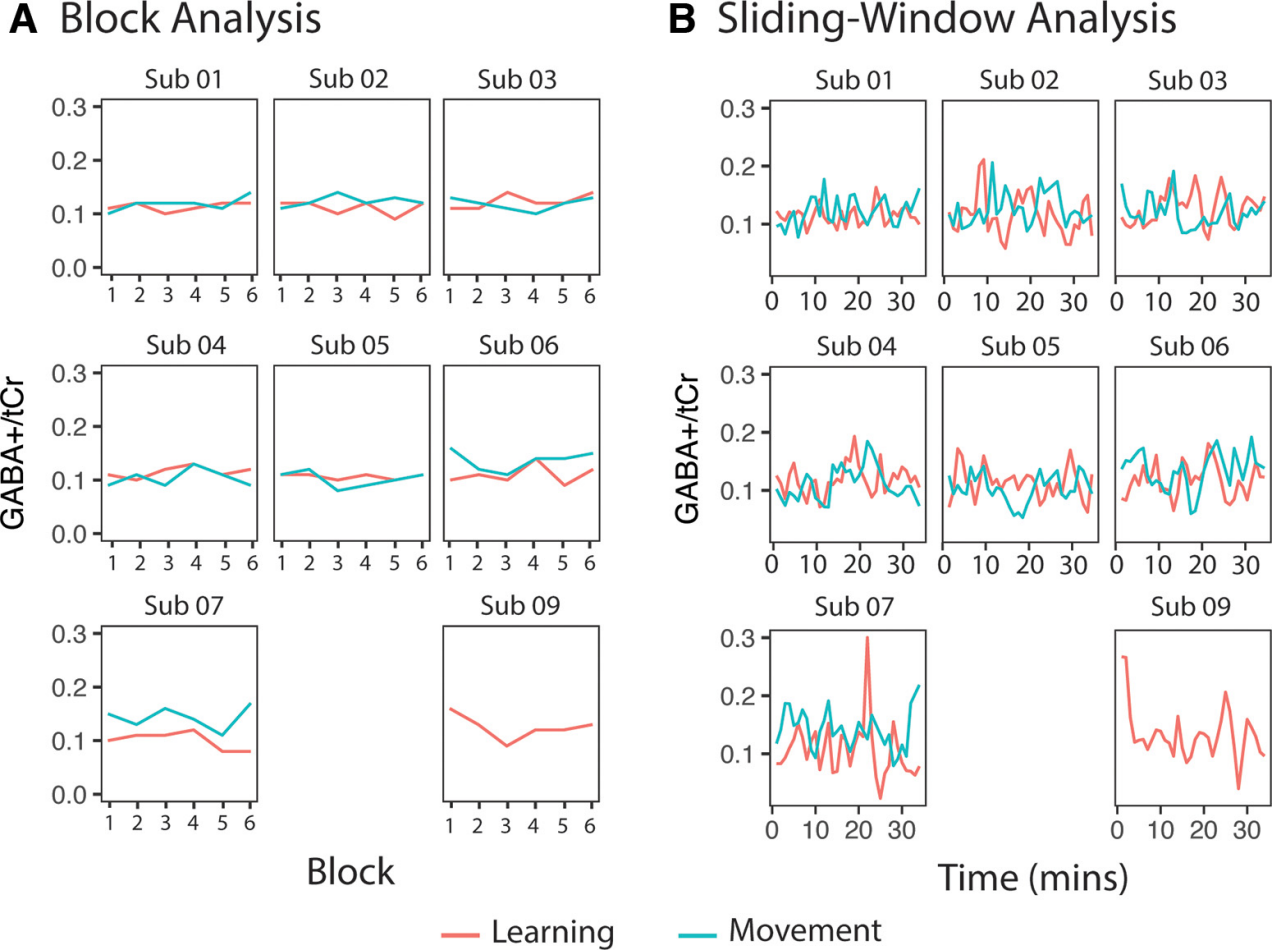
GABA+/tCr levels for each individual participant throughout the task. ***A***, GABA+/tCr levels analyzed using the block analysis. ***B***, GABA+/tCr levels analyzed using the sliding-window analysis. Note: subject (Sub) 08 was removed because of poor data quality. Data from the control task of subject (Sub) 09 was removed because of errors during data acquisition.

Figure 7 shows individual timecourses of GABA+ levels from each participant for each analysis. One subject was removed from GABA analyses because of poor data quality.

#### Block analysis of GABA levels

**Figure 8.**
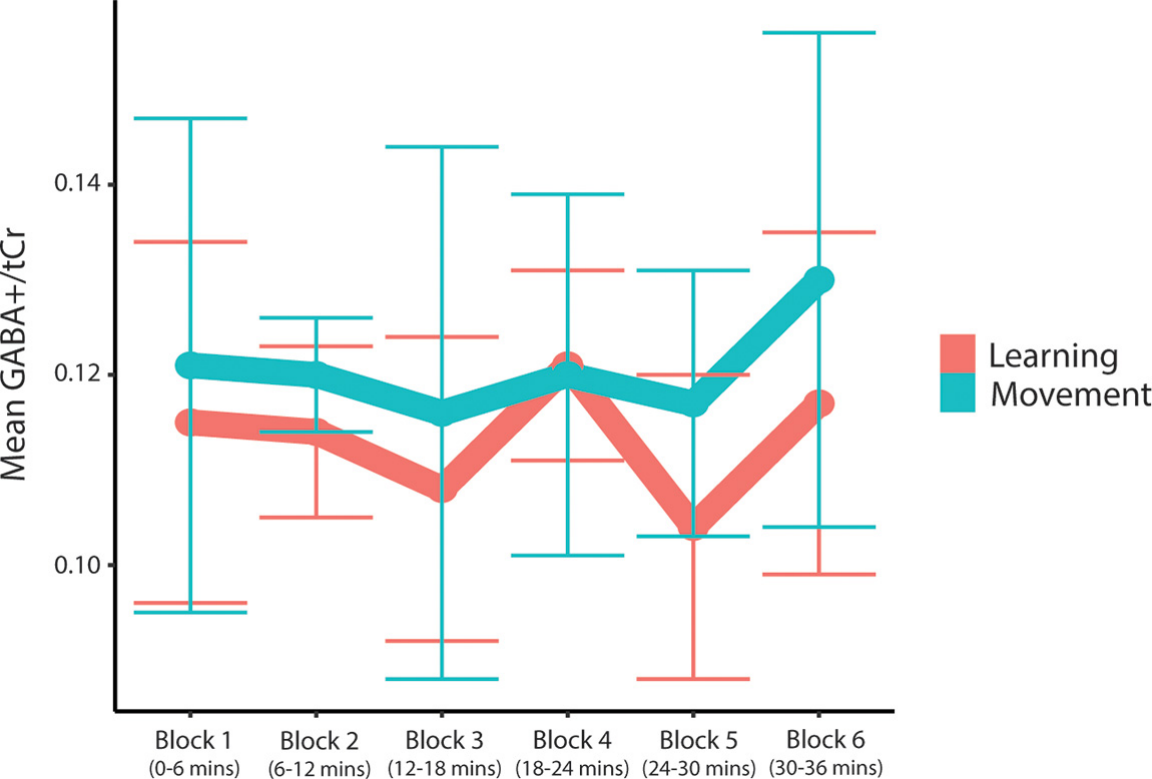
Mean GABA+/tCr levels for each block throughout the task. Error bars represent the SD.

The two-way repeated-measures ANOVA examining GABA+/tCr over time showed no significant effect of condition (*F*(1,6) = 1.6, *p* = 0.26, η^2^ = 0.21), no significant effect of time (*F*(5,30) = 1.8, *p* = 0.14, η^2^ = 0.24), and no significant condition by time interaction effect on GABA+/tCr levels (*F*(5,30) = 0.7, *p* = 0.63, η^2^ = 0.10; Fig. 8). Though block 4 of the learning condition appears not to follow the trend, data quality metrics were consistent across all blocks and at acceptable levels (Fig. 7*A*, individual GABA levels across the tasks, Table 1, mean quality metrics).

For the learning condition, learning was quantified as the percentage change of the median reaction time of block 6 (ending at 36 min) from block 1 (ending at 6 min), denoted as learning block 6. Learning was also quantified in two additional ways according to the methods described in the study by Kolasinski et al. (2019), as follows. (1) The median reaction time from blocks 4–6 (24–36 min) was calculated for each subject, and this was then used to calculate the percentage change from the median reaction time in block 1 (6 min), which is denoted as learning-median; and (2) the percentage change of the block with the lowest median reaction time from block 1, denoted as learning-best block. There were no significant correlations between GABA+/tCr levels in block 1 and learning-block 6, learning-median, or learning-best block. There were also no significant correlations between GABA+/tCr levels in block 6 and learning-block 6.

#### Sliding-window analysis of GABA levels

**Figure 9.**
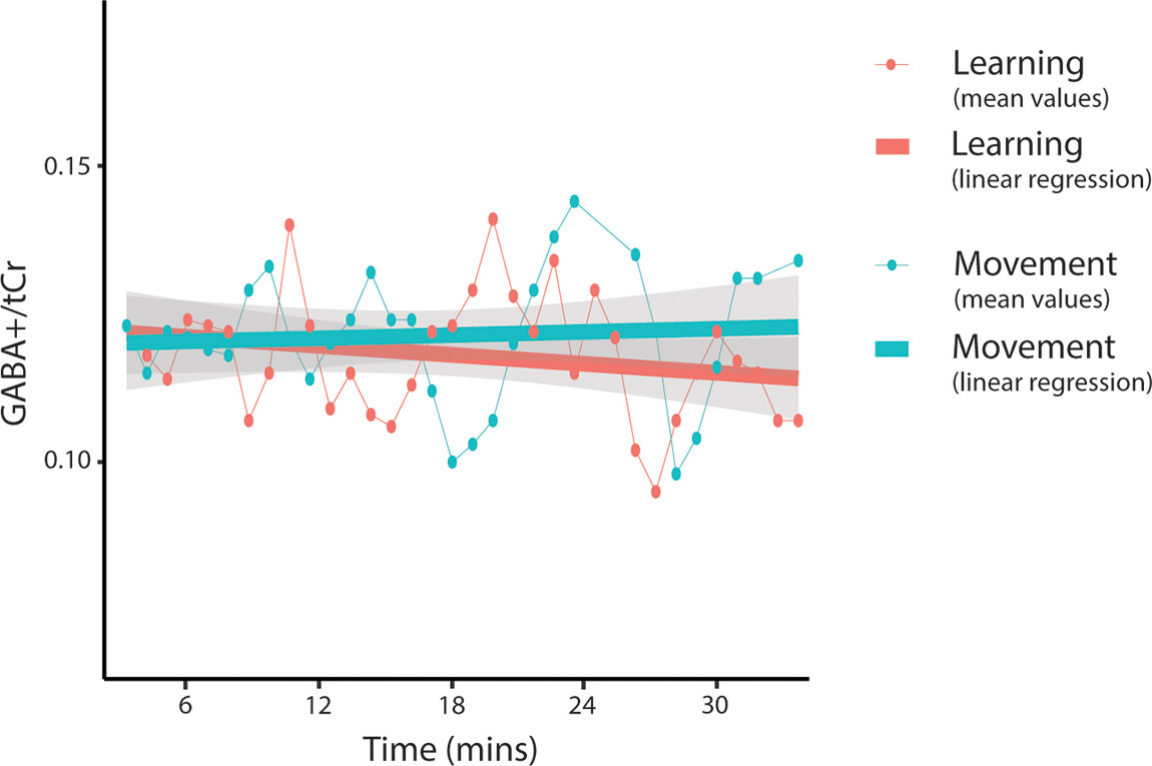
Mean GABA+/tCr levels for each window throughout the task. Dot and thin lines represent the mean of all participants, thick lines represent an estimated linear regression line. Shaded areas represent SE of the estimated regression. MRS data from all subjects for windows 24 (spectra 737–800; ∼22 min) and 25 (spectra 769–832; ∼23 min), and MRS data from three subjects from window 21 (spectra, 641–704; ∼19 min) were removed from the control task because of a NAA linewidth of >13 Hz.

In the sliding-window analysis, neither condition nor time was a significant predictor of GABA+/tCr levels. The condition by time interaction was also not a significant predictor of GABA levels. Including condition as a random effect significantly improved the model (*p* < 0.001), but time as a random effect did not; thus, it was kept as a fixed effect (Fig. 9, Table 3). Adding in time as a quadratic term did not significantly improve the model. As there were no significant interactions, no follow-up analyses were conducted.

### Glutamate

**Figure 10.**
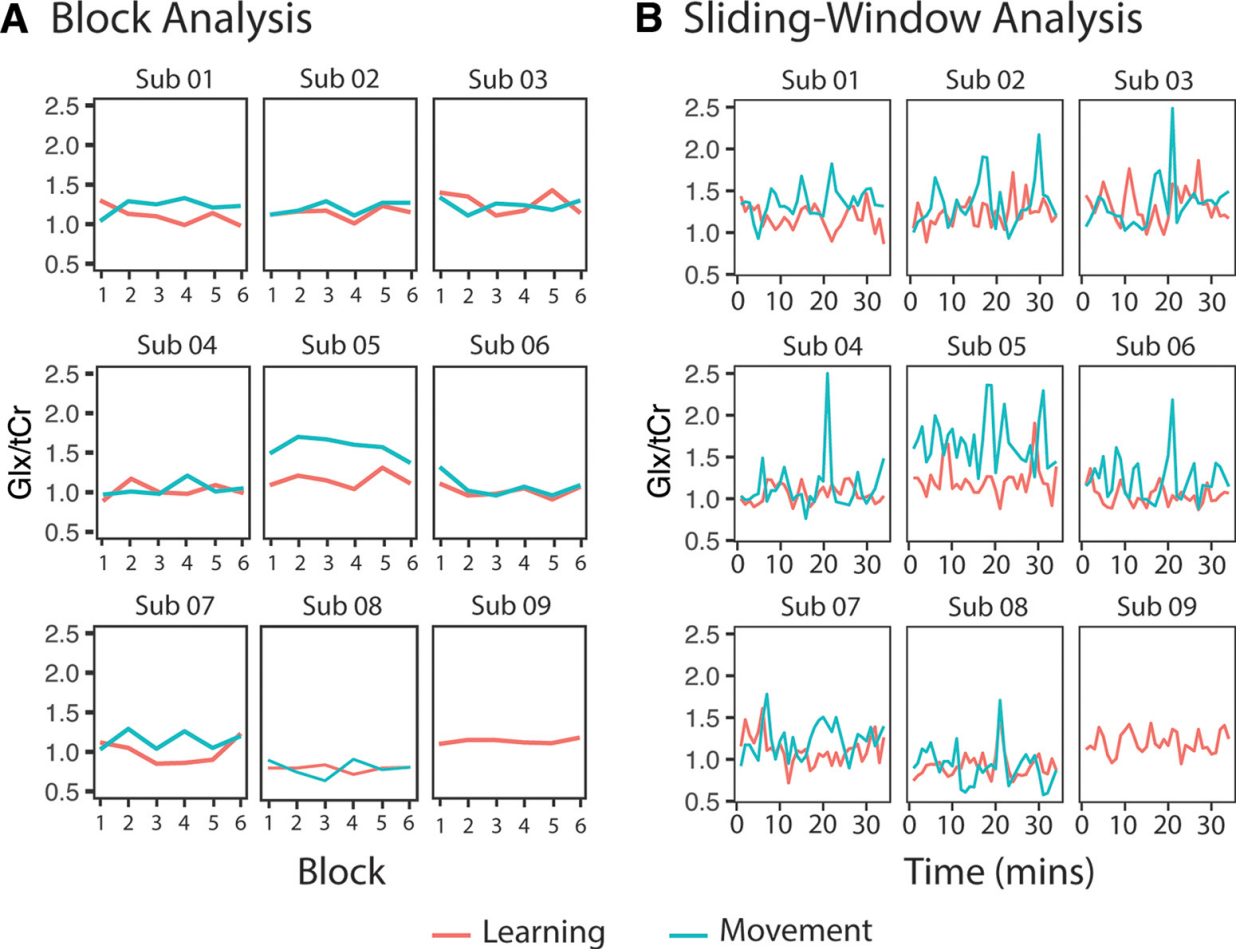
Glx/tCr levels for each individual participant throughout the task. ***A***, Glx/tCr levels analyzed using the block analysis. ***B***, Glx/tCr levels analyzed using the sliding-window analysis. Note: data from the control task of subject (Sub) 09 was removed because of errors during data acquisition.

**Figure 11.**
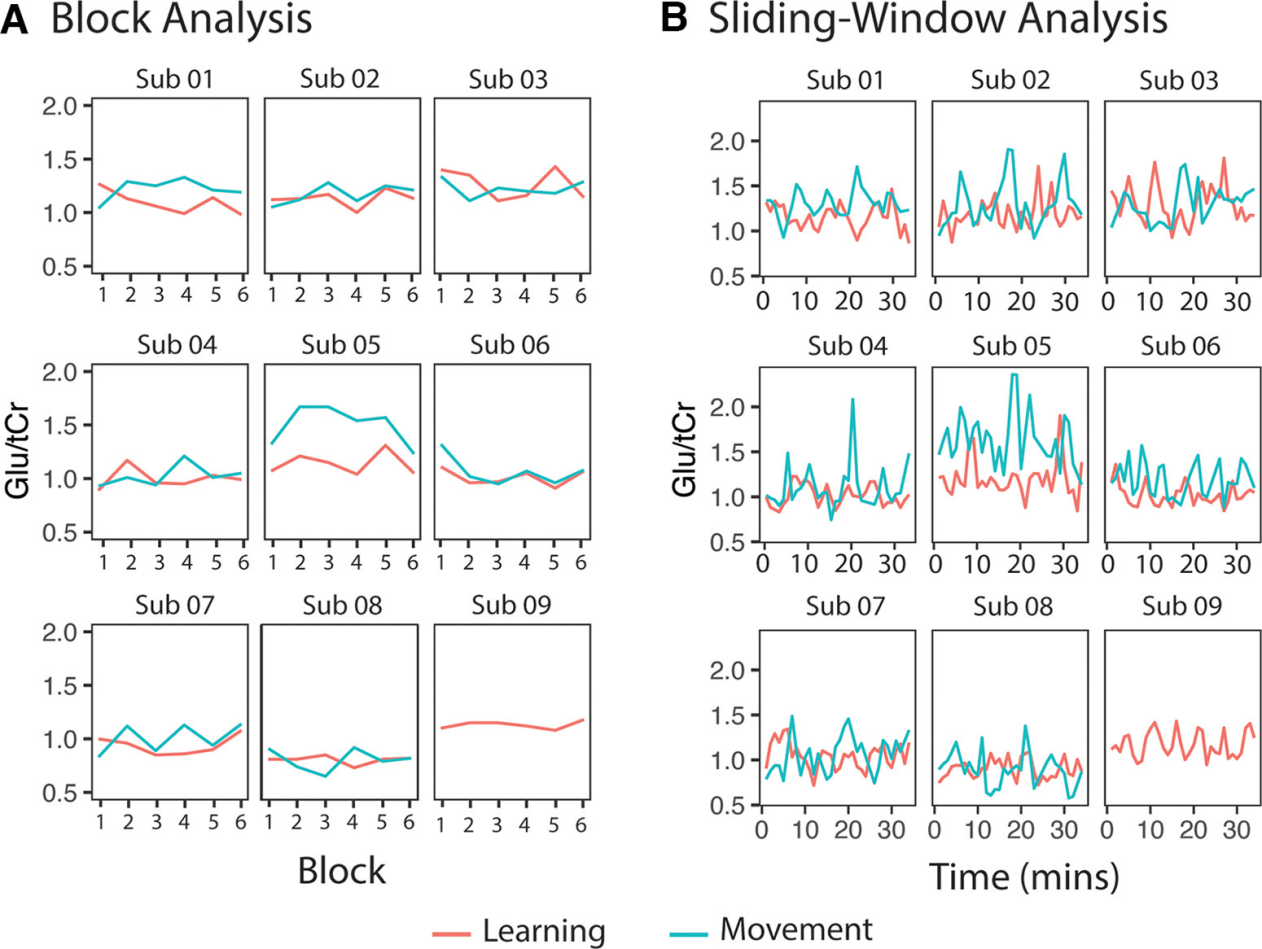
Glu/tCr levels for each individual participant throughout the task. ***A***, Glu/tCr levels analyzed using the block analysis. ***B***, Glu/tCr levels analyzed using the sliding-window analysis. Note: Data from the control task of subject (Sub) 09 was removed because of errors during data acquisition.

Figure 10 shows individual timecourses of Glx/tCr levels from each participant, and Figure 11 shows individual timecourses of Glu/tCr levels from each participant for each analysis.

#### Block analysis of Glx levels

**Figure 12.**
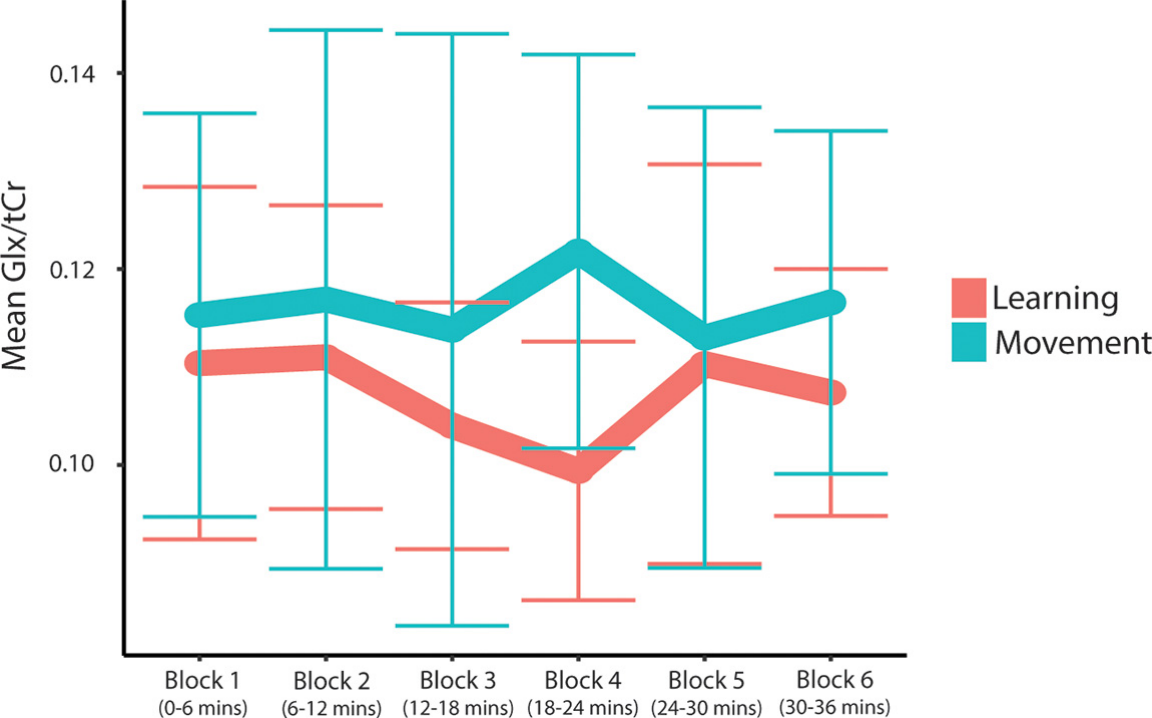
Mean Glx/tCr levels for each block throughout the task. Error bars represent the SD.

The two-way repeated-measures ANOVA examining Glx/tCr over time showed no significant effect of condition (*F*(1,7) = 4.00, *p* = 0.09, η^2^ = 0.36) and no significant effect of time (*F*(5,35) = 0.53, *p* = 0.75, η^2^ = 0.07) on Glx/tCr levels. However, there was a significant time by condition interaction effect on Glx/tCr levels (*F*(5,35) = 2.54, *p* = 0.05, η^2^ = 0.27). Simple main-effects analyses showed no significant effect of time in the learning (*F*(5,40) = 2.11, *p* = 0.17, η^2^ = 0.21, Bonferroni-adjusted *p*-value) or movement condition (*F*(5,35) = 0.67, *p* = 1.0, η^2^ = 0.09, Bonferroni-adjusted *p*-value). In the learning condition, compared with block 1 (ending at 6 min), *post hoc* paired-samples *t* tests showed a significant reduction in Glx/tCr levels at block 4 (ending at 24 min); however, this did not withstand Bonferroni adjustment. There was no significant change in Glx/tCr levels in the movement condition (Fig. 12).

**Figure 13.**
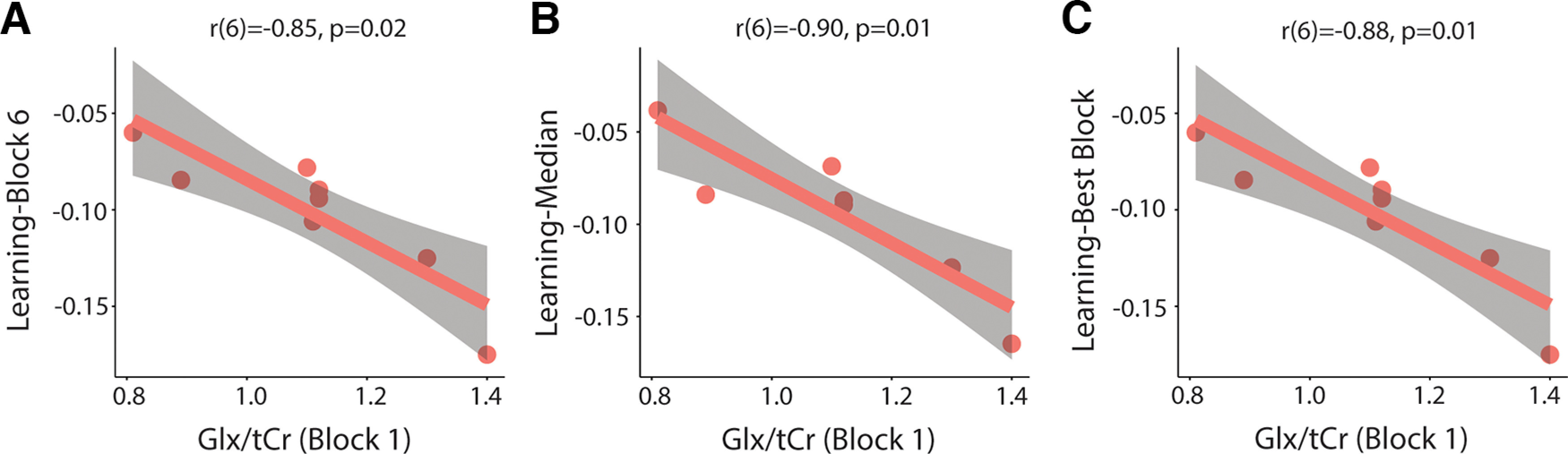
Relationship between levels of Glx/tCr in block 1 and motor learning. ***A***, Significant correlation between block 1 Glx/tCr levels and learning-block 6. ***B***, Significant correlation between block 1 Glx/tCr levels and learning-median. ***C***, Significant correlation between block 1 Glx/tCr levels and learning-best block. All *p*-values are Bonferroni adjusted. Shaded areas represent 95% confidence intervals.

**Figure 14.**
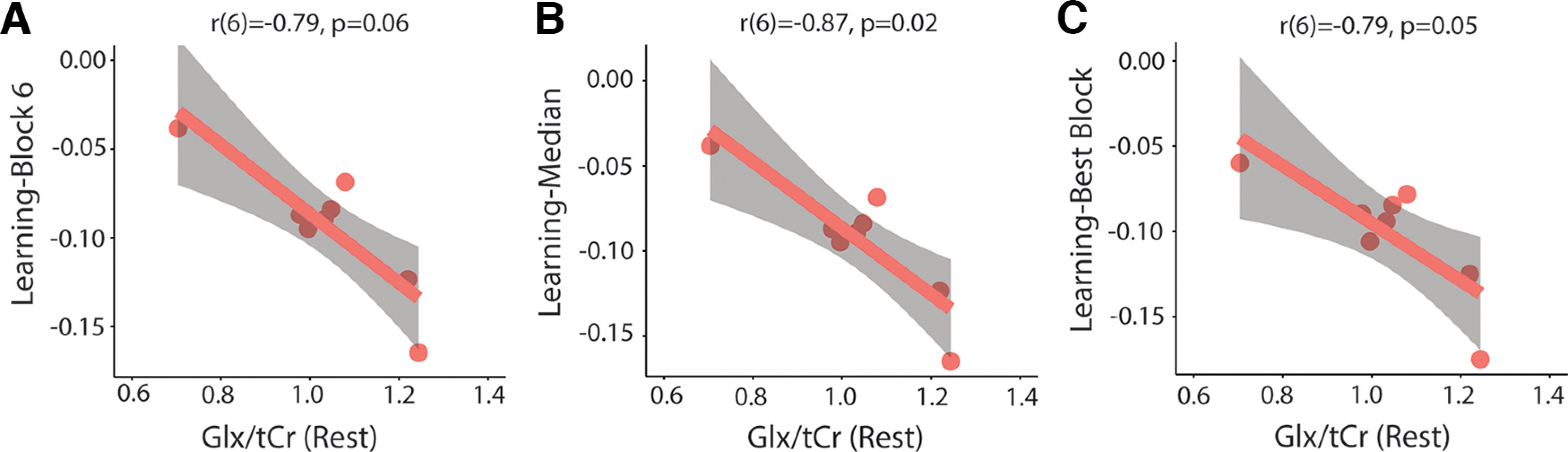
Relationship between levels of Glx/tCr at rest and motor learning. ***A***, Correlation between resting Glx/tCr levels and learning-block 6. ***B***, Significant correlation between resting Glx/tCr levels and learning-median. ***C***, Significant correlation between resting Glx/tCr levels and learning-best block. All *p*-values are Bonferroni adjusted. Shaded areas represent 95% confidence intervals.

There was a significant correlation between Glx/tCr levels in block 1 and learning-block 6 (*r*(6) = −0.85, *p* = 0.02, Bonferroni adjusted *p*-value), learning-median (*r*(6) = −0.90, *p* = 0.007, Bonferroni adjusted *p*-value), and learning-best block (*r*(6) = −0.88, *p* = 0.01, Bonferroni adjusted *p*-value). Higher Glx/tCr levels in block 1 are associated with better motor learning (Fig. 13). Glx/tCr levels in block 6 did not correlate with learning-block 6. Glx/tCr levels at rest also significantly correlated with learning-median (*r*(6) = −0.87, *p* = 0.02, Bonferroni-adjusted *p*-value) and learning-best block (*r*(6) = −0.79, *p* = 0.05, Bonferroni-adjusted *p*-value). There was a significant correlation between Glx/tCr levels at rest and learning-block 6; however, this did not withstand correction for multiple comparisons (*r*(6) = −0.79, *p* = 0.06, Bonferroni-adjusted *p*-value). Higher resting levels of Glx/tCr are associated with better motor learning (Fig. 14).

#### Block analysis of Glu levels

**Figure 15.**
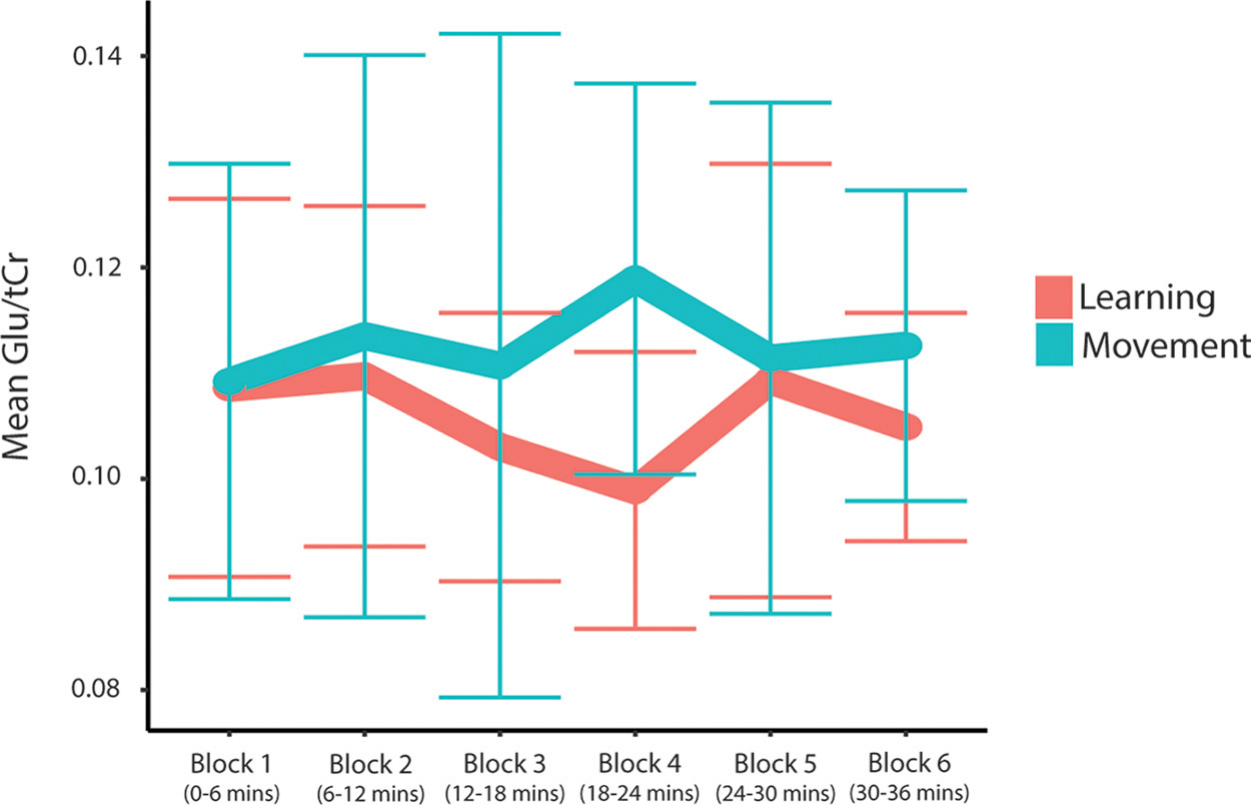
Mean Glu/tCr levels for each block throughout the task. Error bars represent the SD.

The two-way repeated-measures ANOVA examining Glu/tCr over time showed no significant effect of condition (*F*(1,7) = 3.27, *p* = 0.11, η^2^ = 0.32) and no significant effect of time (*F*(5,35) = 0.36, *p* = 0.87, η^2^ = 0.05) on Glu/tCr levels. However, there was a significant time by condition interaction effect on Glu/tCr levels (*F*(5,35) = 2.84, *p* = 0.03, η^2^ = 0.29). Simple main-effects analyses showed no significant effect of time in the learning condition (*F*(5,40) = 2.23, *p* = 0.14, η^2^ = 0.22, Bonferroni-adjusted *p*-value) or movement condition (*F*(5,35) = 0.58, *p* = 1.0, η^2^ = 0.08, Bonferroni-adjusted *p*-value). Compared with block 1 (ending at 6 min), *post hoc* paired-samples *t* tests showed a significant reduction in Glu/tCr levels at block 4 (ending at 24 min); however, this did not withstand Bonferroni adjustment. There was no significant change in Glu/tCr levels in the movement condition (Fig. 15).

**Figure 16.**
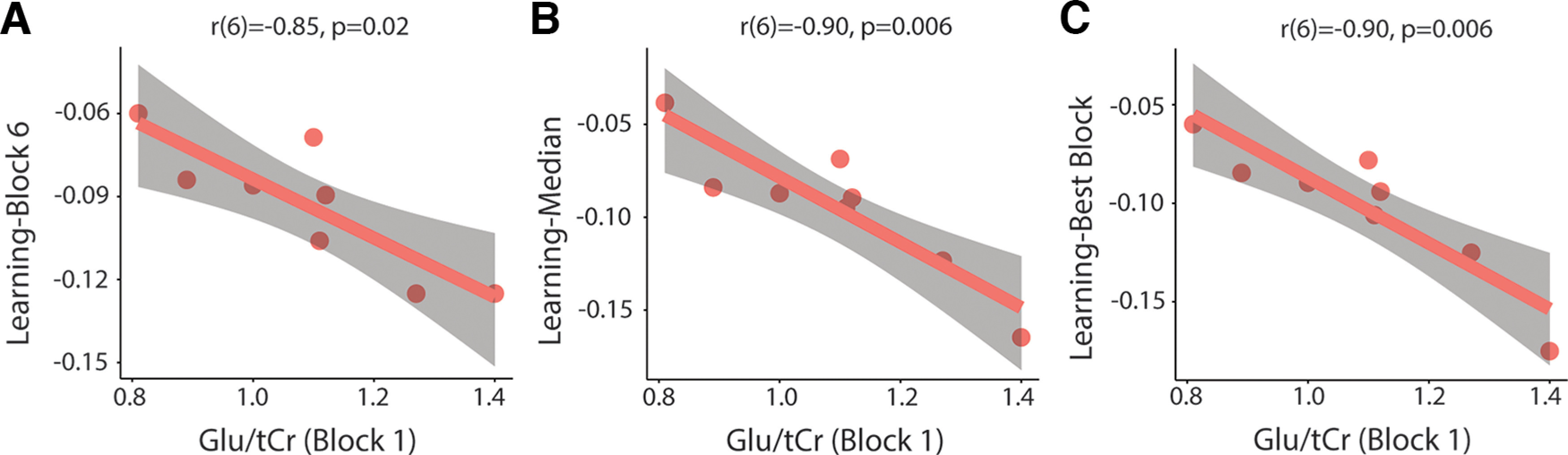
Relationship between levels of Glu/tCr in block 1 and motor learning. ***A***, Significant correlation between block 1 Glu/tCr levels and learning-block 6. ***B***, Significant correlation between block 1 Glu/tCr levels and learning-median. ***C***, Significant correlation between block 1 Glu/tCr levels and learning-best block. All *p*-values are Bonferroni adjusted. Shaded areas represent 95% confidence intervals.

**Figure 17.**
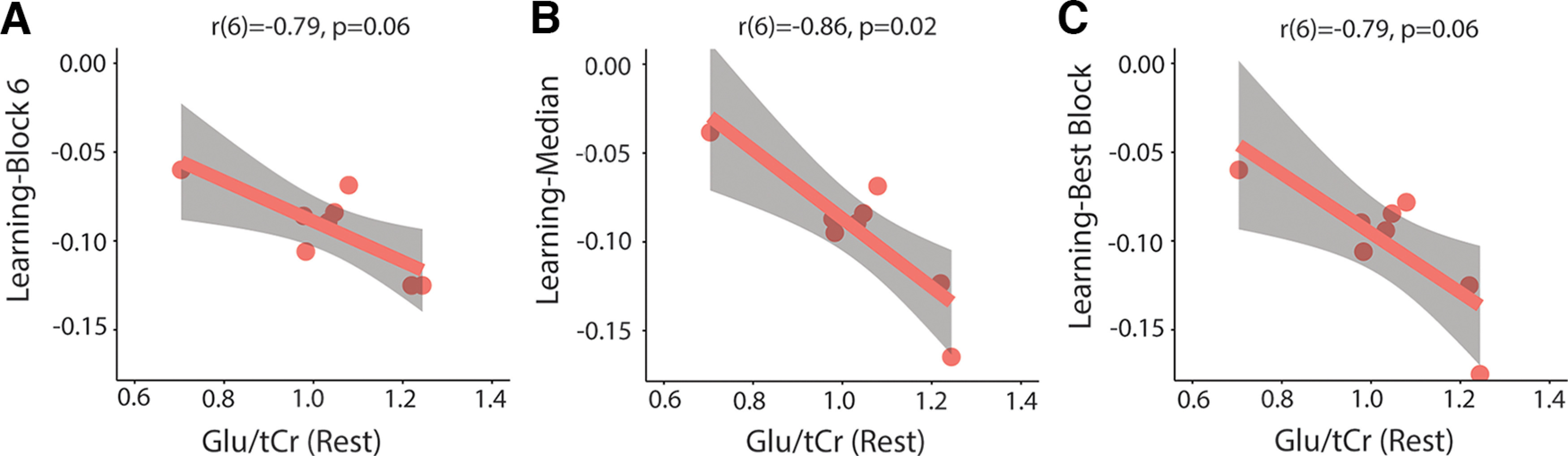
Relationship between levels of Glu/tCr at rest and motor learning. ***A***, Correlation between resting Glu/tCr levels and learning-block 6. ***B***, Significant correlation between resting Glu/tCr levels and learning-median. ***C***, Correlation between resting Glu/tCr levels and learning-best block. All *p*-values are Bonferroni adjusted. Shaded areas represent 95% confidence intervals.

Consistent with the Glx results, there was a significant correlation between Glu/tCr levels in block 1 and learning-block 6 (*r*(6) = −0.85, *p* = 0.02, Bonferroni-adjusted *p*-value), learning-median (*r*(6) = −0.90, *p* = 0.006, Bonferroni-adjusted *p*-value), and learning-best block (*r*(6) = −0.90, *p* = 0.006, Bonferroni-adjusted *p*-value). Higher Glu/tCr levels in block 1 are associated with better motor learning (Fig. 16). Glu/tCr levels in block 6 did not correlate with learning-block 6. A similar trend was seen for resting levels of Glu/tCr. Glu/tCr levels at rest significantly correlated with learning-median (*r*(6) = −0.86, *p* = 0.02, Bonferroni-adjusted *p*-value). Glu/tCr levels at rest also correlated with learning-best block and learning-block 6; however, these did not withstand correction for multiple comparisons (learning-best block: *r*(6) = −0.79, *p* = 0.06, Bonferroni-adjusted *p*-value; learning-block 6: *r*(6) = −0.79, *p* = 0.06, Bonferroni-adjusted *p*-value; Fig. 17).

#### Sliding-window analysis of Glx levels

**Figure 18.**
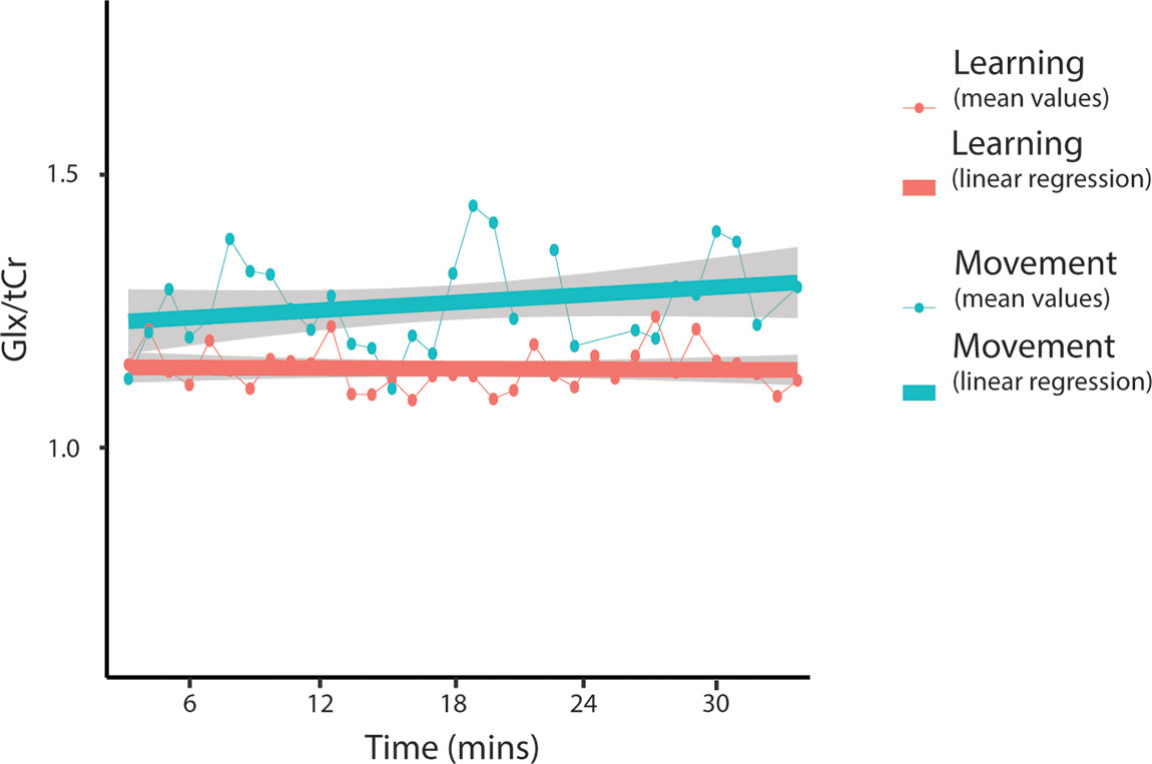
Mean Glx/tCr levels for each window throughout the task. Dot and thin lines represent the mean of all participants, thick lines represent an estimated linear regression line. Shaded areas represent the SE of the estimated regression. MRS data from all subjects for windows 24 (spectra 737–800; ∼22 min) and 25 (spectra 769–832; ∼23 min), and MRS data from three subjects from window 21 (spectra 641–704; ∼19 min) were removed from the control task because of a NAA linewidth of >13 Hz.

Neither condition nor time were significant predictors of Glx/tCr levels. The condition by time interaction was also not a significant predictor of Glx levels. Including condition as a random effect significantly improved the model (*p* < 0.001), but time as a random effect did not; thus, it was kept as a fixed effect (Fig. 18, Table 4). Adding in time as a quadratic term did not significantly improve the model. As there were no significant interactions, no follow-up analyses were conducted.

**Table 3:** Summary of linear mixed effects model parameters from the sliding-window analysis of GABA+/tCr

Parameter	Fixed effects					Random effects	
	Estimate	SE	95% CI	*t*	*p*	Variance	SD
Intercept	0.12	0.004	0.11, 0.13	27.73	*p* < 0.001	0.00003	0.006
Condition	−0.003	0.008	−0.02, 0.01	−0.46	0.656	0.0003	0.02
Time	−0.0002	0.0002	−0.0006, 0.0001	−1.19	0.235		
Condition by time	0.0003	0.0003	−0.0003, 0.0009	1.07	0.287		

95% CIs were approximated using the Wald method.

#### Sliding-window analysis of Glu levels

**Figure 19.**
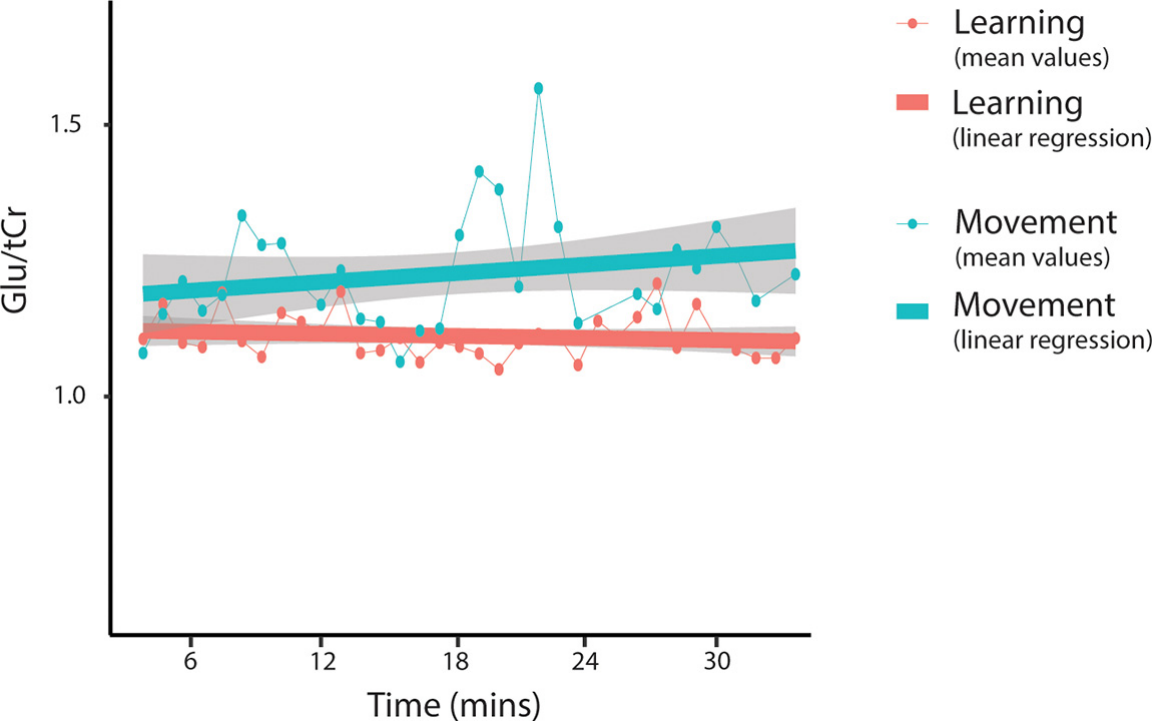
Mean Glu/tCr levels for each window throughout the task. Dot and thin lines represent the mean of all participants, thick lines represent an estimated linear regression line. Shaded areas represent SE of the estimated regression. MRS data from all subjects for windows 24 (spectra 737–800; ∼22 min) and 25 (spectra 769–832; ∼23 min), and MRS data from three subjects from window 21 (spectra 641–704; ∼19 min) were removed from the control task because of a NAA linewidth of >13 Hz.

Neither condition nor time was a significant predictor of Glu/tCr levels. The condition by time interaction was also not a significant predictor of Glu levels. Including condition as a random effect significantly improved the model (*p* < 0.001), but time as a random effect did not; thus, it was kept as a fixed effect (Fig. 19, Table 5). Adding in time as a quadratic term did not significantly improve the model. As there were no significant interactions, no follow-up analyses were conducted.

**Table 4:** Summary of linear mixed effects model parameters from the sliding window analysis of Glx/tCr

Parameter	Fixed effects					Random effects	
	Estimate	SE	95% CI	*t*	*p*	Variance	SD
Intercept	1.15	0.04	1.05, 1.24	25.90	*p* < 0.01	0.01	0.11
Condition	0.09	0.06	−0.03, 0.22	1.63	0.13	0.02	0.13
Time	−0.0002	0.001	−0.003, 0.002	−0.13	0.90		
Condition by time	0.003	0.002	−0.0004, 0.0067	1.74	0.08		

95% CIs were approximated using the Wald method.

**Table 5:** Summary of linear mixed effects model parameters from the sliding window analysis of Glu/tCr

Parameter	Fixed effects					Random effects	
	Estimate	SE	95% CI	*t*	*p*	Variance	SD
Intercept	1.12	0.04	1.05, 1.24	26.10	*p* < 0.01	0.01	0.11
Condition	0.07	0.06	−0.03, 0.22	1.35	0.20	0.02	0.13
Time	−0.0006	0.001	−0.003, 0.002	−0.53	0.60		
Condition by time	0.003	0.002	−0.0004, 0.0067	1.75	0.08		

95% CIs were approximated using the Wald method.

## Discussion

Here we use MEGA-PRESS at 3 T to measure changes in GABA and glutamate levels during a motor learning task compared with a control task with no learning (movement condition). Using both block and sliding-window analyses over time, no significant changes in GABA+/tCr, Glx/tCr, or Glu/tCr levels were found in either task. However, Glx/tCr and Glu/tCr levels at rest and at the start of the task were related to learning later in the task.

Our findings of no change in GABA+/tCr levels are in contrast to those of Kolasinski et al. (2019) and Floyer-Lea et al. (2006), who found a significant decrease in GABA levels specifically during motor learning. This may be because of several reasons. First, the voxel size used in the present study (2.5 × 2.5 × 2.5 cm^3^) was larger than that used in the previous studies (2.0 × 2.0 × 2.0 cm^3^). The SNR in the MRS spectra is proportional to the main magnetic field strength, the volume of the voxel, and the number of signal averages (Mikkelsen et al., 2018a). The increase in voxel size was used to offset the lower SNR because of the lower magnetic field strength (3 T vs the 7 T strength used by Kolasinski et al., 2019) and reduced number of signal averages included in the sliding-window analysis. However, the larger voxel size results in partial volume effects (i.e., the inclusion of tissue outside of the motor cortex), which may have impacted our ability to detect metabolite changes. Second, the magnitude of motor learning may be related to the magnitude of GABA changes. Though we showed a statistically significant drop in reaction time in the learning group only, the mean change in reaction time was ∼50 ms in the present study, whereas participants in the study by Kolasinski et al. (2019) reduced their reaction time by ∼100 ms on average. The reason for this discrepancy is unclear as both studies included samples with similar demographics, though compliance and motivation are possible contributors. Though Kolasinski et al. (2019) found no significant relationship between the magnitude of the change in GABA/tCr and the magnitude of learning, they hypothesize that the reduction in GABA/tCr may not scale linearly with learning. Third, the present study used “Gannet” (Edden et al., 2014) to analyze the 3 T GABA MRS data, whereas the previous studies used “LCModel” (Provencher, 1993). Analysis software has substantial impact on the resulting metabolite values (Craven et al., 2022), and it is possible that Gannet may be less sensitive to small changes than LCModel. Further, a recent conference abstract shows no correlation between GABA values measured at 7 T and analyzed using LCModel and GABA values measured at 3 T and analyzed using Gannet (Bell et al., 2022). A contributing factor to these differences between the software is how each handles macromolecules. LCModel can be configured to attempt to mitigate the influence of macromolecules, whereas Gannet does not estimate macromolecular contribution, it simply assumes a 50% contribution across all data. Though the macromolecular signal is assumed to be functionally irrelevant, its contribution to the GABA+ signal varies (Harris et al., 2015b), and there is evidence that its contribution can confound behavioral relationships. Mikkelsen et al. (2018b) showed stronger correlations between vibrotactile behavior and GABA levels with the macromolecule signal suppressed compared with the same relationship with GABA+ data. This likely has less of an impact on the current data because of its functional nature; however, functional changes in the macromolecule signal have yet to be explored.

We found no significant changes in Glx or Glu levels in either analysis. This is in agreement with Kolasinski et al. (2019) and Floyer-Lea et al. (2006), who found no changes in glutamate levels during either motor learning or a control movement task using a block analysis. However, this is in contrast to the findings of Chen et al. (2017), who found changes in glutamate (and GABA) levels during a hand-clenching task using sliding-window fMRS at 7 T, though hand-clenching is different in nature than button pressing. Chen et al. (2017) also used a smaller voxel compared with the present study, and GABA was measured using an editing sequence that suppresses the macromolecule signal. A macromolecular-suppressed technique was not used in the present study because of the lower SNR. Finally, in contrast to the present study where glutamate was quantified using the OFF sub-spectra, Chen et al. (2017) quantified glutamate from the difference spectra, which produces a substantially different glutamate result (Bell et al., 2020).

We found a positive relationship between motor learning and glutamate levels both at rest and at the start of the task. Higher glutamate levels in block 1 were associated with a larger reduction in reaction time later in the task. We found no relationship between GABA levels and motor learning. In contrast, Kolasinski et al. (2019) found no relationship between glutamate levels and motor learning, but did see a relationship between GABA levels and motor learning. Lower levels of GABA in block 1 were related to a greater reduction in reaction time later in the task. This was hypothesized to represent disinhibition of the motor cortex, a theory that aligns with our findings. Disinhibition of the motor cortex allows for an increase in excitation, represented here as the higher glutamate levels. Indeed, there is evidence that MRS measured glutamate levels correlate with cortical excitability (Stagg et al., 2011), therefore participants with higher resting/block 1 glutamate levels may begin the task with higher cortical excitability. Studies in humans have shown that increasing cortical excitability using noninvasive brain stimulation enhances motor learning (Reis and Fritsch, 2011).

The difference in findings may be because of the choice of acquisition echo time. Kolasinski et al. (2019) used a short echo time (TE = 36 ms), whereas the present study used a longer echo time to allow for the editing pulses (TE = 68 ms). At TE = 36 ms, the intensity of the overlapping glutamine peaks is high, making it harder to distinguish between glutamate and glutamine. As glutamine is both a precursor and a breakdown product of glutamate, functional changes in glutamate may be masked by functional changes in glutamine. Using simulations, Mullins et al. (2008) showed that the intensity of the 2.35 ppm glutamine C-4 peak is substantially attenuated around TE = 70 ms in relation to glutamate in the same area,. Therefore, it is possible that there is less glutamine contaminant in the signal acquired at TE = 68 ms than in the TE = 36 ms study. However, at 7 T the peaks are more easily resolved because of increased spectral resolution, which will also reduce glutamine contaminant. The differences in findings may also be because of partial volume effects. Because of the larger voxel size in this study, it is likely that our voxel contains more signal from the supplementary motor area, which may be the source of the relationship between glutamate and motor learning. In contrast, this extra signal from outside areas may mask the more subtle, motor cortex-specific GABA relationship.

At time point 20, there is a spike in the linewidth of the control (movement) task, and the control task overall has higher linewidth than the learning task. The spike could be a fatigue effect, in that participants are becoming bored and beginning to move around that time; however, if this was the case it would be expected that the FWHM would remain high. Similarly, the difference in FWHM between tasks may be because of more movement in the control task. As participants are told to respond as fast as possible and there will be no “learning” in the control task, this may cause more movement as they attempt to react quickly. As the cause of this spike is not obvious, we have been as transparent as possible with our quality parameters to show that this is not caused by bad quality data.

A limitation of this study is the small sample size. Power analyses were calculated based on data obtained at 7 T. 7 T data has improved signal-to-noise ratio, and it is therefore possible that a change of a similar magnitude would be harder to detect at 3 T. However, GABA-edited MRS was used in the current study. While 7 T has a much greater signal, GABA remains overlapped by the more abundant creatine signal; thus, the differences in GABA signal between GABA-edited MRS at 3 T and nonedited MRS at 7 T study are not completely clear. In addition, the effect size reported by Kolasinski et al. (2019) is much higher than typically reported in fMRS studies. For example, a review by Mullins (2018) found the average change in glutamate levels to be 7%. Though we assumed a more conservative effect size for power analysis, it is likely that further replication of this study may identify the true effect size to be smaller. Mikkelsen et al. (2018a) showed a minimum sample size of six is needed to detect a 20% change in sensorimotor GABA levels when averaging 64 transients and Nezhad et al. (2020) showed a minimum sample size of eight is needed to detect a 15% change in sensorimotor GABA levels using a within-session design, though both were based on a larger voxel than used in the present study. Power analyses determined a sample size of 14 participants would be needed to detect a smaller effect size of 0.1.

Another limitation of our study was the large voxel size used, resulting in signal from the sensorimotor cortex as well as from the motor cortex. A larger voxel size is needed to offset the low SNR of GABA-edited spectra; however, this will reduce the specificity of our results. Our findings show that this may be a particular issue with functional studies, where it is important to acquire signal from a specific region. Functional studies may need to consider the tradeoff of a slower temporal resolution compared with a larger voxel size. Future fMRS studies may find it beneficial to use a short echoplanar imaging (EPI) sequence to map the functional region, to determine whether to prioritize a smaller voxel over a shorter acquisition period (Carlson et al., 2017).

In conclusion, using MEGA-PRESS at 3 T, we found no significant changes in GABA+/tCr, Glx/tCr, or Glu/tCr levels in either a motor learning or control task. We demonstrate a positive relationship between motor learning and glutamate levels both at rest and at the start of the task, and hypothesize this to represent higher cortical excitability, in line with findings from the literature. Our study highlights some of the issues facing the fMRS literature and can be used as a foundation for future studies investigating metabolite levels in motor learning.

## Synthesis

Reviewing Editor: Satu Palva, Helsingin yliopisto Decisions are customarily a result of the Reviewing Editor and the peer reviewers coming together and discussing their recommendations until a consensus is reached. When revisions are invited, a fact-based synthesis statement explaining their decision and outlining what is needed to prepare a revision will be listed below. The following reviewer(s) agreed to reveal their identity: NONE.

This manuscript addresses an important question in assessing analysis methods for fMRS of GABA at 3 T. In particular, the authors wish to validate sliding window analysis for MEGA-PRESS at 3 T and replicate reported changes at 7 T during a motor learning task. There is a significant rationale behind this work as fMRS-type studies are being conducted at 3 T. The authors do not find any significant relationship between time-course of glutamate or GABA during motor task or learning which they attribute to sequence differences compared to previous studies.

Major

The diagram showing the timing of epochs, and the corresponding timing of MRS analysis steps is appreciated, but a diagram outlining/presenting the behavioral task should also be included.

The sample size was gained from a power calculation based on previous literature values of the effect size seen in a similar task, however the previous work was performed at 7T which brings with it improved signal to noise. This fact should be addressed, as it is likely the study is underpowered with the current number of subjects, given the lower SNR at 3T compared to 7T. Reference to the work of Sanaei et al (Nezhad, F. S., LeaCarnall, C. A., Anton, A., Jung, J., Michou, E., Williams, S. R., & Parkes, L. M. (2020). Number of subjects required in common study designs for functional GABA magnetic resonance spectroscopy in the human brain at 3 Tesla. European Journal of Neuroscience, 51(8), 1784-1793. https://doi.org/10.1111/ejn.14618) which may have helped guide power calculations, should also be made and considered.

Similarly the choice of a 1800 ms TR will reduce SNR due to T1 effects. Some discussion of the reason for the choice made here should be made. It is true, 1800 ms will give greater temporal resolution, but as all data is either binned at 64 ms, or 5 minutes, temporal resolution may not be as important in this situation.

The use of Mullins et al (2008) as a reference to suggest a TE of 68 ms would allow better separation of glutamate and glutamine is incorrect, 68 ms was not tested in that study, with 40, and 80 ms being found to prehaps be best to allow reliable glutamate measurement at 3T. Note Hancu and Port (2011) also suggest a TE of 80 ms provides reliable measures of glutamate and glutamine, but this does not suggest 68 ms is likewise better than a TE of 36 ms. The ability to seperate the two metabolites is a result of changes in spectral patterns due to J coupling evolution of the spectra, which should be calculated, or considered when selecting a TE. Indeed, this is why a TE of 68 ms is considered optimal for MEGA-press for GABA.

When discussing the time windows that were removed from the sliding window analysis results in the control period, (page 11, lines 240 - 241) rather than present as 22 minutes, 23 minutes etc, I would suggest the use of "minute 22, Minute 23" etc to define the time points missing. This is because one reading may suggest 22 minutes of data was removed, rather than approximately 1 minute.

The last line fo the Abstract states "Though further refinement and validation of this method is needed, this is the first step in using functional MRS at 3T to probe GABA levels in both healthy cognition and clinical disorders." This is an incorrect statement. Other fMRS studies investigating GABA changes in response to stimuli have already been performed. Please correct. I would suggest "this study represents a further step..."

Similarly the significance statement proposes that this study "is a foundational study for future studies investigating GABA and glutamate levels simultaneously during motor learning." I'm not sure I would be so strong in my statements. In addition they miss out several other actual foundational studies in GABA fMRS (e.g. Sanae et al) and so may want check the literature a bit more deeply. Especially if they wish this paper to be of use to future GABA fMRS researchers.

Line 31: Glutamate might not be relatively easily detected at 3 T with a standard PRESS sequence. In particular, glutamate overlaps considerably with glutamine at 3 T which makes detection challenging (Tkac et al. 2001) and requires dedicated sequences. In fact, this is also stated in Line 152. The authors report 'glutamate' as glutamate and also glutamate + glutamine (Glx) because of this.

Further to that point. In the discussion (Line 404): It is implied that the longer TE choice of the current study should produce more reliable glutamate than previous studies (Kolsinksi et al.) carried out at 7 T. It is true that intensity of either peak will be lower, but at 7 T the glutamine peak should be better resolved due to improved spectral resolution. Whereas the present 3 T work glutamate is largely expected to be glutamate+glutamine, therefore the 3 T measures reported in this work may be more sensitive to glutamine changes. The authors go on to say that 'This is supported by the consistency of Glx and glutamate' - this does not seem to be clear evidence to support their statements since it may be because any observed change is driven by glutamine rather than glutamate. Could the authors comment on this?

Tkac, I., Andersen, P., Adriany, G., Merkle, H., Uurbil, K., & Gruetter, R. (2001). In vivo 1H NMR spectroscopy of the human brain at 7 T. Magnetic Resonance in Medicine, 46(3), 451-456. https://doi.org/10.1002/MRM.1213

Regarding the linewidth changes (to assess BOLD effect on spectra) the authors decide to use an existing tool to automate measurement. However, it is unclear how this is carried out - is this on the group averaged data? If so, I think that by measuring linewidth on the group averaged data there is a danger of losing sensitivity to any small changes on a participant-basis. In addition, it is unclear how group averaging is performed - this is not straight forward for MRS since data needs to be aligned and scaled accordingly. Potentially in that process linewidth broadening/narrowing can occur. Could the authors comment on that and elaborate on the methodology. I would recommend analysing the subject-specific linewidth changes comparing the rest period to stimulation period.

There seems to be a spike in the data at time-point 20 visible in the Glu timecourse (of several subjects (S03/4/6/8) and the FWHM plot. What is happening here? There also appears to be some data points missing. There also looks like there could be a consistent difference in FWHM between learning and control tasks? If this the case - why? I would expect both data quality to be the same.

Can the authors also ensure all the parameters in terms of acquisition and data quality, as laid out by the recent MRSinMRS consensus paper, have been reported.

Lin, A., Andronesi, O., Bogner, W., Choi, I. Y., Coello, E., Cudalbu, C., Juchem, C., Kemp, G. J., Kreis, R., Krssak, M., Lee, P., Maudsley, A. A., Meyerspeer, M., Mlynarik, V., Near, J., Oz, G., Peek, A. L., Puts, N. A., Ratai, E. M., ... Mullins, P. G. (2021). Minimum Reporting Standards for in vivo Magnetic Resonance Spectroscopy (MRSinMRS): Experts' consensus recommendations. NMR in Biomedicine, 34(5), e4484. https://doi.org/10.1002/NBM.4484

One of the main arguments in the discussion for the negative finding in this study was the voxel size and partial volume effect. Could the authors include an averaged overlay image from all participants showing the voxel positioning in this study. This would help future studies when planning voxels in the motor region at 3 T.

Does the Gannet pipeline include tissue correction? Could the authors report tissue content in each voxel and how much it varies across participants?

Could the authors include an example fit alongside the representative data shown in the manuscript.

Minor:

Abstract, first line: The comparison as stated doesn't make sense since the difficulty in measuring GABA at 3 T is same for fMRS or conventional MRS. Both require edited sequence and sufficient SNR.

Line 371: I welcome the discussion point but would be cautious about using the (Bell et al. 2022) reference regarding 3 T vs. 7 T GABA since this is only a conference abstract.

Line 419 - 'needed to offset the low SNR of the GABA signal' - specifically this should be the low SNR of MEGA-edited GABA spectra. Reference to the work of Sanaei et al

## Author Response

This manuscript addresses an important question in assessing analysis methods for fMRS of GABA at 3 T. In particular, the authors wish to validate sliding window analysis for MEGA-PRESS at 3 T and replicate reported changes at 7 T during a motor learning task. There is a significant rationale behind this work as fMRS-type studies are being conducted at 3 T. The authors do not find any significant relationship between time-course of glutamate or GABA during motor task or learning which they attribute to sequence differences compared to previous studies.

We thank the reviewer for their comments and in recognising the importance of this manuscript. We have included detailed responses to their comments below.

Major

(1) The diagram showing the timing of epochs, and the corresponding timing of MRS analysis steps is appreciated, but a diagram outlining/presenting the behavioral task should also be included.

As this is a registered report, the introduction and methods cannot be altered from the approved stage 1 submission. Depending on the guidance from the editorial team, we are willing to include a new figure (see below) as this is not a change in the methodology per se but to provide additional clarification to the text but need direction as to whether this is indeed appropriate.

(2) The sample size was gained from a power calculation based on previous literature values of the effect size seen in a similar task, however the previous work was performed at 7T which brings with it improved signal to noise. This fact should be addressed, as it is likely the study is underpowered with the current number of subjects, given the lower SNR at 3T compared to 7T. Reference to the work of Sanaei et al (Nezhad, F. S., Lea‐Carnall, C. A., Anton, A., Jung, J., Michou, E., Williams, S. R., & Parkes, L. M. (2020). Number of subjects required in common study designs for functional GABA magnetic resonance spectroscopy in the human brain at 3 Tesla. European Journal of Neuroscience, 51(8), 1784-1793. https://doi.org/10.1111/ejn.14618) which may have helped guide power calculations, should also be made and considered.

This has been addressed in the discussion (page 18) and the paper cited.

Page 19: A limitation of this study is the small sample size. Power analyses were calculated based on data obtained at 7T. 7T data has improved signal to noise and it is therefore possible that a change of a similar magnitude would be harder to detect at 3T. However, GABA-edited MRS was used in the current study. While 7T has much greater signal, GABA remains overlapped by the more abundant Creatine signal, thus the differences in GABA signal between GABA-edited MRS at 3T and non-edited MRS at 7T study are not completely clear. In addition, the effect size reported by Kolasinski et al. (2019) is much higher than typically reported in fMRS studies. For example, a review by Mullins (2018) found the average change in glutamate levels to be 7%. Though we assumed a more conservative effect size for power analysis, it is likely that further replication of this study may identify the true effect size to be smaller. Mikkelsen et al. (2018) showed a minimum sample size of 6 is needed to detect a 20% change in sensorimotor GABA levels when averaging 64 transients and Nezhad et al. (2020) showed a minimum sample size of 8 is needed to detect a 15% change in sensorimotor GABA levels using a within-session design, though both were based on a larger voxel than used in the present study. Power analyses determined a sample size of 14 participants would be needed to detect a smaller effect size of 0.1.

(3) Similarly the choice of a 1800 ms TR will reduce SNR due to T1 effects. Some discussion of the reason for the choice made here should be made. It is true, 1800 ms will give greater temporal resolution, but as all data is either binned at 64 ms, or 5 minutes, temporal resolution may not be as important in this situation.

A TR of 1800 ms was chosen based on previous GABA studies (e.g. Brix et al., 2017; Cleve et al., 2017). Whilst we agree with the reviewer there will be some signal loss, this increases the number of transients that we acquire within the 6-minute bin. To our knowledge the trade-off between the signal loss vs the increased number of transients in edited functional MRS has not been explored. As this method is not uncommon and was accepted at stage one, we did not feel it was necessary to focus on it in the discussion.

(4) The use of Mullins et al (2008) as a reference to suggest a TE of 68 ms would allow better separation of glutamate and glutamine is incorrect, 68 ms was not tested in that study, with 40, and 80 ms being found to prehaps be best to allow reliable glutamate measurement at 3T. Note Hancu and Port (2011) also suggest a TE of 80 ms provides reliable measures of glutamate and glutamine, but this does not suggest 68 ms is likewise better than a TE of 36 ms. The ability to seperate the two metabolites is a result of changes in spectral patterns due to J coupling evolution of the spectra, which should be calculated, or considered when selecting a TE. Indeed, this is why a TE of 68 ms is considered optimal for MEGA-press for GABA.

Though the reviewer is correct that TE=68 ms was not specifically tested in the Mullins 2008 paper, simulations were conducted for TE=60 ms and TE=70 ms, as well as TE=30 ms and TE=40 ms. The glutamine signal intensity is substantially lower at TE=70 ms vs TE=40 ms, though we did not intend to suggest that this means a TE=68 ms creates a “better” measure, only to highlight the differences in signal. We have rephrased this section to better clarify.

Page 18: Using simulations, Mullins et al. (2008) showed that the intensity of the 2.35 ppm glutamine C-4 peak is substantially attenuated around TE=70 ms in relation to glutamate in the same area,. Therefore, it is possible that there is less glutamine contaminant in signal acquired at TE=68 ms than in the TE=36 ms study.

(5) When discussing the time windows that were removed from the sliding window analysis results in the control period, (page 11, lines 240 - 241) rather than present as 22 minutes, 23 minutes etc, I would suggest the use of “minute 22, Minute 23” etc to define the time points missing. This is because one reading may suggest 22 minutes of data was removed, rather than approximately 1 minute.

We have updated the manuscript accordingly.

(6) The last line for the Abstract states “Though further refinement and validation of this method is needed, this is the first step in using functional MRS at 3T to probe GABA levels in both healthy cognition and clinical disorders.” This is an incorrect statement. Other fMRS studies investigating GABA changes in response to stimuli have already been performed. Please correct. I would suggest “this study represents a further step...”

We have updated the abstract as suggested

(7) Similarly the significance statement proposes that this study “is a foundational study for future studies investigating GABA and glutamate levels simultaneously during motor learning.” I’m not sure I would be so strong in my statements. In addition they miss out several other actual foundational studies in GABA fMRS (e.g. Sanae et al) and so may want check the literature a bit more deeply. Especially if they wish this paper to be of use to future GABA fMRS researchers.

We have updated the significance statement to remove the term foundational and we have expanded the citations to include other foundational works as suggested while working to maintain a concise discussion.

(8) Line 31: Glutamate might not be relatively easily detected at 3 T with a standard PRESS sequence. In particular, glutamate overlaps considerably with glutamine at 3 T which makes detection challenging (Tkac et al. 2001) and requires dedicated sequences. In fact, this is also stated in Line 152. The authors report ‘glutamate’ as glutamate and also glutamate + glutamine (Glx) because of this.

Further to that point. In the discussion (Line 404): It is implied that the longer TE choice of the current study should produce more reliable glutamate than previous studies (Kolsinksi et al.) carried out at 7 T. It is true that intensity of either peak will be lower, but at 7 T the glutamine peak should be better resolved due to improved spectral resolution. Whereas the present 3 T work glutamate is largely expected to be glutamate+glutamine, therefore the 3 T measures reported in this work may be more sensitive to glutamine changes. The authors go on to say that ‘This is supported by the consistency of Glx and glutamate’ - this does not seem to be clear evidence to support their statements since it may be because any observed change is driven by glutamine rather than glutamate. Could the authors comment on this?

Tkác, I., Andersen, P., Adriany, G., Merkle, H., Uurbil, K., & Gruetter, R. (2001). In vivo 1H NMR spectroscopy of the human brain at 7 T. Magnetic Resonance in Medicine, 46(3), 451-456. https://doi.org/10.1002/MRM.1213

We apologize for not detailing the nuances in the glutamate and glutamine signals; as the reviewer notes, this is complicated and much of this (on going) work is beyond the scope of the current study. As introduced in response to comment 4, we suggest highly suppressed glutamine signal at 68 ms means it is unlikely that any changes seen would be due to glutamine, as these changes would be very small. We did not intend to imply that TE=68ms was a better measure and have rephrased this section accordingly.

Page 18: Using simulations, Mullins et al. (2008) showed that the intensity of the glutamine peak is lower at TE=70 ms than at TE=30 ms, therefore it is possible that there is less glutamine contaminant in signal acquired at TE=68. However at 7T, the peaks are more easily resolved due to increased spectral resolution, which will also reduce glutamine contaminant.

(9) Regarding the linewidth changes (to assess BOLD effect on spectra) the authors decide to use an existing tool to automate measurement. However, it is unclear how this is carried out - is this on the group averaged data? If so, I think that by measuring linewidth on the group averaged data there is a danger of losing sensitivity to any small changes on a participant-basis. In addition, it is unclear how group averaging is performed - this is not straight forward for MRS since data needs to be aligned and scaled accordingly. Potentially in that process linewidth broadening/narrowing can occur. Could the authors comment on that and elaborate on the methodology. I would recommend analysing the subject-specific linewidth changes comparing the rest period to stimulation period.

We used the same procedure described in the methods section to measure linewidth changes, however instead of subtracting the spectra (group average functional - group average baseline), we used the existing tool to measure the difference between the linewidth of the group average functional block and the group average baseline. This has been clarified and more detail added.

Page 10: We would like to report a minor change to the analyses. To determine the effects of the BOLD signal on linewidth, we originally stated that we would subtract the group averaged resting data from the group averaged functional data for each block. Instead we used the tool op_matchLW from the FID-A toolbox (Simpson et al., 2017) to measure the difference in linewidth between the group averaged resting data from the group averaged functional data for each block, removing any subjectivity from the procedure. Data were aligned prior to averaging using the op_alignAverages function. Scripts for this procedure can be found at [URL redacted for double-blind review].

We chose to compare group averaged data based on common practice in the literature (e.g. Betina Ip et al., 2017; Mangia et al., 2007). Though we agree with the reviewer that using individual subject data to measure linewidth changes may capture different changes, this would be a substantial a change from our previously approved methodology.

(10) There seems to be a spike in the data at time-point 20 visible in the Glu timecourse (of several subjects (S03/4/6/8) and the FWHM plot. What is happening here? There also appears to be some data points missing. There also looks like there could be a consistent difference in FWHM between learning and control tasks? If this the case - why? I would expect both data quality to be the same.

Regarding the spike at timepoint 20, we are unsure what has caused this, and therefore were hesitant to speculate. We have included some theories on page 20.

Page 18: At timepoint 20 there is a spike in the linewidth of the control (movement) task, and the control task overall has higher linewidth than the learning task. The spike could be a fatigue effect, in that participants are becoming bored and beginning to move around that time, however if this was the case it would be expected that the FWHM would remain high. Similarly, the difference in FWHM between tasks may be due to more movement in the control task. As participants are told to respond as fast as possible and there will be no “learning” in the control task, this may cause more movement as they attempt to react quickly. As the cause of this spike is not obvious, we have been as transparent as possible with our quality parameters to show this is not caused by bad quality data.

Regarding the missing data points, MRS data from all subjects for windows 24 (spectra 737-800, roughly 22 minutes) and 25 (spectra 769-832, roughly 23 minutes) and MRS data from three subjects from window 21 (spectra 641-704, roughly 19 minutes) were removed from the control task due to a NAA linewidth of greater than 13 Hz. This has been reiterated in the figure legends where data is missing (See Figures 9, 18 and 19).

(11) Can the authors also ensure all the parameters in terms of acquisition and data quality, as laid out by the recent MRSinMRS consensus paper, have been reported.

Lin, A., Andronesi, O., Bogner, W., Choi, I. Y., Coello, E., Cudalbu, C., Juchem, C., Kemp, G. J., Kreis, R., Krššák, M., Lee, P., Maudsley, A. A., Meyerspeer, M., Mlynarik, V., Near, J., Öz, G., Peek, A. L., Puts, N. A., Ratai, E. M., ... Mullins, P. G. (2021). Minimum Reporting Standards for in vivo Magnetic Resonance Spectroscopy (MRSinMRS): Experts’ consensus recommendations. NMR in Biomedicine, 34(5), e4484. https://doi.org/10.1002/NBM.4484

We have updated the manuscript to include information on how we calculated linewidth and SNR and have reported the fit error (GABA) and CRLB (glutamate). Regarding the acquisition parameters, the reported parameters have been accepted as part of the registered report and the methods section cannot be altered. Again, based on guidance from the editor, we can include these details if this is modification acceptable.

Page 11: Linewidth and SNR were calculated using tools from the FID-A toolbox. Linewidth was calculated as the full width at half maximum of the NAA peak using the tool op_getLW . SNR was calculated as the amplitude of the NAA peak (1.8 - 2.2 ppm) divided by the standard deviation of the noise (-2 - 0 ppm) using the tool op_getSNR. Table 2 and Figure 3 show fit metrics averaged over the entire group for the block and sliding window analyses, respectively.

Figure 3: Quality metrics for sliding window analysis. (A) Mean NAA Full Width Half Maximum (FWHM) in Hz. (B) Mean NAA Signal to Noise Ratio (SNR). Error bars represent standard deviation. (C) Mean GABA Fit Error (%) calculated in Gannet. (D) Glutamate Cramer-Rao Lower Bound (CRLB) calculated in LCModel.

(12) One of the main arguments in the discussion for the negative finding in this study was the voxel size and partial volume effect. Could the authors include an averaged overlay image from all participants showing the voxel positioning in this study. This would help future studies when planning voxels in the motor region at 3 T.

We have included an averaged overly as part of figure 2.

Figure 2: (A) Example of GABA data from the block analysis (number of signal averages (NSA)=184). (B) Example of glutamate data obtained from the off sub-spectra from the block analysis (NSA=184). (C) Example of GABA data from the sliding window analysis (NSA=64). (D) Example of glutamate data obtained from the off sub-spectra from the sliding window analysis (NSA=64). (E) Heatmap of voxel placement, yellow shows areas of high overlap.

(13) Does the Gannet pipeline include tissue correction? Could the authors report tissue content in each voxel and how much it varies across participants?

The Gannet pipeline can include tissue correction; however it was not applied in this study for two reasons. First, tissue correction is not typically applied when referencing to a metabolite (we referenced to Creatine in this study). Second, this study used within-subject statistics, therefore metabolites were compared within the same voxel at different timepoints, rather than between subjects. Therefore, alterations in tissue content between subjects would not affect the overall outcome. We have included the tissue content below for the reviewer but have not included this in the manuscript due to the above reason. Additionally, the voxel placement figure shows high overlap between voxels.

(14) Could the authors include an example fit alongside the representative data shown in the manuscript.

We have included an example fit with the example data in figure 2 (see response to comment 12)

Minor:

Abstract, first line: The comparison as stated doesn’t make sense since the difficulty in measuring GABA at 3 T is same for fMRS or conventional MRS. Both require edited sequence and sufficient SNR.

This has been rephrased to: Functional magnetic resonance spectroscopy (fMRS) of GABA at 3T poses additional challenges compared to functional MRS of other metabolites due to the difficulties of measuring GABA levels.

Line 371: I welcome the discussion point but would be cautious about using the (Bell et al. 2022) reference regarding 3 T vs. 7 T GABA since this is only a conference abstract.

We have altered this sentence to highlight that it is a conference abstract.

Further, a recent conference abstract shows no correlation between GABA values measured at 7T and analysed using LCModel and GABA values measured at 3T and analysed using Gannet (Bell et al., 2022).

Line 419 - ‘needed to offset the low SNR of the GABA signal’ - specifically this should be the low SNR of MEGA-edited GABA spectra.

We have updated the manuscript with this change.
